# Resource diversity disturbs marine *Vibrio* diversity and community stability, but loss of *Vibrio* diversity enhances community stability

**DOI:** 10.1002/ece3.11234

**Published:** 2024-04-19

**Authors:** Xinyi Qin, Qinghua Hou, Huaxian Zhao, Pengbin Wang, Shu Yang, Nengjian Liao, Jiongqing Huang, Xiaoli Li, Qing He, Rajapakshalage Thashikala Nethmini, Gonglingxia Jiang, Shiying He, Qingxiang Chen, Ke Dong, Nan Li

**Affiliations:** ^1^ Laboratory for Coastal Ocean Variation and Disaster Prediction, Key Laboratory of Climate, Resources and Environment in Continental Shelf Sea and Deep Sea of Department of Education of Guangdong Province, College of Ocean and Meteorology Guangdong Ocean University Zhanjiang China; ^2^ Key Laboratory of Environment Change and Resources use in Beibu Gulf, Ministry of Education Nanning Normal University Nanning China; ^3^ Key Laboratory of Marine Ecosystem Dynamics, Second Institute of Oceanography Ministry of Natural Re‐Sources Hangzhou China; ^4^ College of Environmental Science and Engineering Guilin University of Technology Guilin China; ^5^ School of Agriculture Ludong University Yantai China; ^6^ Department of Biological Sciences Kyonggi University Suwon‐si Gyeonggi‐do South Korea

**Keywords:** 16S rRNA gene, assembly process, community stability, resource diversity, *Vibrio*

## Abstract

*Vibrio* is a salt‐tolerant heterotrophic bacterium that occupies an important ecological niche in marine environments. However, little is known about the contribution of resource diversity to the marine *Vibrio* diversity and community stability. In this study, we investigated the association among resource diversity, taxonomic diversity, phylogenetic diversity, and community stability of marine *Vibrio* in the Beibu Gulf. *V. campbellii* and *V. hangzhouensis* were the dominant groups in seawater and sediments, respectively, in the Beibu Gulf. Higher alpha diversity was observed in the sediments than in the seawater. Marine *Vibrio* community assembly was dominated by deterministic processes. Pearson's correlation analysis showed that nitrite (${\mathrm{NO}}_{2}^{-}$‐N), dissolved inorganic nitrogen (DIN), ammonium (${\mathrm{NH}}_{4}^{+}$‐N), and pH were the main factors affecting marine *Vibrio* community stability in the surface, middle, and bottom layers of seawater and sediment, respectively. Partial least‐squares path models (PLS‐PM) demonstrated that resource diversity, water properties, nutrients, and geographical distance had important impacts on phylogenetic and taxonomic diversity. Regression analysis revealed that the impact of resource diversity on marine *Vibrio* diversity and community stability varied across different habitats, but loss of *Vibrio* diversity increases community stability. Overall, this study provided insights into the mechanisms underlying the maintenance of *Vibrio* diversity and community stability in marine environments.

## Graphical abstract

That the impact of resource diversity on marine *Vibrio* diversity and community stability varied in different habitats, but an increase in taxonomic and phylogenetic diversity weakened community stability.

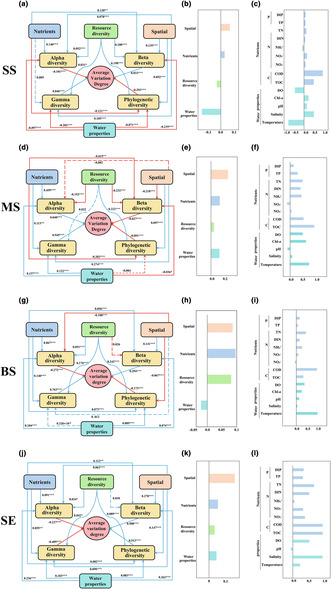

## 1 INTRODUCTION

Clarifying the association between diversity and community stability is a core issues in ecology (Donohue et al., 2013). The term biodiversity includes taxonomic and phylogenetic diversity (Cadotte et al., 2012; Vellend, 2003). Taxonomic diversity refers to the richness and abundance of species (Craven et al., 2018), whereas phylogenetic diversity describes the diversity of evolutionary lineages (Cadotte et al., 2012). Community stability is defined as the capacity to withstand environmental disturbances (Shade et al., 2012, 2013). In recent decades, the relationship between diversity and community stability has been studied extensively (Craven et al., 2018; Erkus et al., 2013; McCann, 2000; McNaughton, 1988; Wagg et al., 2022). For example, Jiao et al. (2019) suggested that, as alpha diversity increases, bacterial communities become more stable in terrestrial ecosystems. In contrast, Yang et al. (2023) revealed that the loss of microbial diversity enhances community stability in soil ecosystems. These studies demonstrate that the relationship between biodiversity and community stability is complex and varies in heterogeneous habitats.

Heterotrophic bacteria are prevalent in the marine ecosystem and exhibit high taxonomic and phylogenetic diversity (Hou et al., 2015; Torsvik et al., 2002). *Vibrio*, a typical heterotrophic bacterium with salt tolerance that is widely distributed in marine environments, plays a crucial role in marine food webs and nutrient cycling (Farmer et al., 2015; Moriarty, 1998; Thompson, Iida, & Swings, 2004; Westrich, 2015). *Vibrio* species, such as *V. cholerae*, *V. parahaemolyticus*, and *V. vulnificus*, can be pathogenic to aquatic animals and humans via the consumption of contaminated seafood (Chiang & Chuang, 2003; Colwell & Spira, 1992; Guin et al., 2019). Marine *Vibrio* taxonomic and phylogenetic diversity studies have recently received increased attention (Kopprio et al., 2020; Vezzulli et al., 2009; Xu et al., 2021). For example, Chen et al. (2020) reported that there were significant differences in marine *Vibrio* diversity across different seasons in the Beibu Gulf. Wang et al. (2019) discovered significant differences in taxonomic diversity among various sea areas. Environmental factors (e.g., water properties and nutrients) can regulate the marine *Vibrio* diversity (Chen et al., 2020; Siboni et al., 2016; Urdaci et al., 1988; Xu et al., 2021). Takemura et al. (2014) conducted a meta‐analysis and found that dissolved organic carbon (DOC) has a strong impact on the marine *Vibrio* community. Xu et al. (2020) observed that the marine *Vibrio* taxonomic diversity is driven by temperature, dissolved oxygen (DO), nitrate (${\mathrm{NO}}_{3}^{-}$‐N), and nitrite (${\mathrm{NO}}_{2}^{-}$‐N) in Dongshan Bay. Li et al. (2020) identified ammonium (${\mathrm{NH}}_{4}^{+}$‐N) and dissolved inorganic phosphorus (DIP) as key factors affecting the community structure of marine *Vibrio* in eutrophic and oligotrophic groups in the Beibu Gulf, respectively. The ecological evolutionary and biogeographical that affect the maintaining mechanism of marine microorganisms are highly complex (Sichert & Cordero, 2021), and the biodiversity and community stability of various habitats are sustained by multiple factors (Sichert & Cordero, 2021). Despite advancements in our understanding of marine *Vibrio* diversity, the relationship between diversity and community stability and its response to changes in the ecological niche remains unclear.

Community assembly not only affects diversity but also indirectly affects community stability (Chase et al., 2011). Microbial community assembly is simultaneously influenced by stochastic and deterministic processes (Zhou & Ning, 2017). Deterministic processes emphasise the abiotic environment (i.e. environmental filtration) and biotic interactions within communities (Zhou & Ning, 2017). In contrast, stochastic processes emphasise unpredictable events such as birth, death, dispersal, and colonisation (Li et al., 2020). The distribution of marine *Vibrio* is influenced by both deterministic and stochastic processes (Jesser Kelsey et al., 2018). Li et al. (2020) demonstrated that the most important process for the assembly of the marine free‐living *Vibrio* community in the Beibu Gulf is stochastic process. Currently, there is a lack of information regarding how community assembly affects *Vibrio* diversity and community stability in marine habitats.

The Beibu Gulf is a semi‐enclosed bay located in the northwest of the South China Sea, and a large number of estuaries and frequent human activities have resulted in abundant nutritional resources, which resulting in imbalanced environmental characteristics and an uneven distribution of resources (Chen et al., 2009; Lai et al., 2014). To explore the relationship among the resource diversity, taxonomic diversity, phylogenetic diversity, and community stability of marine *Vibrio*, we analysed samples from different layers of seawater and sediment from the Beibu Gulf using high‐throughput sequencing of the *Vibrio*‐specific 16S rRNA gene (Liang et al., 2019; Siboni et al., 2016) and aimed to reveal the (i) community structure and assembly process of marine *Vibrio* in different habitats, (ii) influence of resource diversity on taxonomic and phylogenetic diversity, and (iii) key drivers affecting the relationship between marine *Vibrio* diversity and community stability in different habitats. We hypothesised that resource diversity influences the taxonomic and phylogenetic diversity of marine *Vibrio* and community stability in the subtropical marginal sea.

## 2 MATERIALS AND METHODS

### 2.1 Sampling sites and environmental parameters

The sampling sites were located in the Beibu Gulf near Guangxi Province (Figure S1). In total, 405 samples from 25 sites were collected from seawater at various depths and sediments during open cruises of the Beibu Gulf on 10 August 2021. Seawater was sampled from each site at two to three depths ranging from 3 to 40 m, and the sediment samples were divided into four categories: the surface layer of seawater (SS), the middle layer of seawater (MS), the bottom layer of seawater (BS), and sediment (SE) (Figure S1). The SS, MS, BS, and SE groups comprised 125, 85, 110, and 85 samples, respectively. At each site, five replicate water samples were collected using a 5‐L sterile bucket. All seawater samples were stored at 4°C before *Vibrio* isolation and the analysis of environmental factors. Sediment replicates were collected from each site using a 0.05‐m^2^ van Veen grab and pooled together before treatment. All sediments replicates were stored at 20°C on board and 80°C in the laboratory until DNA extraction. For *Vibrio* community analysis in seawater, a vacuum pump was used to sequentially filter 1 L of seawater per sample through 3‐μm filters (Port Washington, NY, USA) to remove debris and larger organisms, and the resulting samples were collected on 0.22‐μm pore size polycarbonate membranes (Millipore Corporation, Billerica, MA, USA). The environmental parameters of the samples were analysed. A portable metre (556 MPS; YSI, USA) was used to determine the temperature, salinity, pH, and DO. And the concentrations of phosphate (${\mathrm{PO}}_{4}^{3-}$‐P), ${\mathrm{NO}}_{2}^{-}$‐N, ${\mathrm{NO}}_{3}^{-}$‐N, and ${\mathrm{NH}}_{4}^{+}$‐N were measured by continuous flow analyser (Seal‐AA3, Germany). Referring to previous methods for determining the chlorophyll *a* (Chl‐*a*) (American Public Health Association, 1926). Use the TOC‐VCPH analyser (Shimadzu, Japan) to determine the amount of total organic carbon (TOC). The chemical oxygen demand (COD) was measured using the alkaline KMnO4 method. DIN was indicated by the sum of ${\mathrm{NO}}_{2}^{-}$‐N, ${\mathrm{NO}}_{3}^{-}$‐N, and ${\mathrm{NH}}_{4}^{+}$‐N, while DIP was represented by the ${\mathrm{PO}}_{4}^{3-}$‐P.

### 2.2 DNA extraction and PCR amplification

Genomic DNA of seawater extraction was performed by a DNeasy PowerWater Kit (QIAGEN, USA) and 0.22‐μm pore size polycarbonate membranes according to the manufacturer's protocols. Sediment DNA was extracted from each sediment sample (0.5 g) using PowerSoil DNA Isolation Kits (Mo Bio Laboratories, Inc., Carlsbad, CA, USA). DNA yield and purity were evaluated using a Nanodrop‐2000 Spectrophotometer (Thermo Scientific, USA). The DNA sample was preserved at −80°C. The regions of the *Vibrio*‐specific 16S rDNA were amplified using primers 169F (5′‐GGCGTAAAGCGCATGCAGGT‐3′) and 680R (5′‐GAAATTCTACCCCCCTCTACAG‐3′) (Thompson, Randa, et al., 2004). A total reaction volume of 20 μL of PCR mixture containing 2 μL of DNA template, 6 μL of ddH_2_O, 10 μL of 2 × Taq PCR Mastermix (TianGen, China), and 2 μL of forward and reverse primers was used. Using a Bio‐Rad thermocycler (Hercules, CA, USA), the amplification process was as follows: an initial activation step at 94°C for 1 min, followed by 35 cycles of denaturation at 95°C for 30 s, annealing at 56°C for 30 s, and extension at 72°C for 30 s, and a final elongation step at 72°C for 10 min. Ultrapure water was used as a negative control instead of the sample solution to rule out the possibility of false‐positive PCR results. PCR products were verified using 2% agarose gel electrophoresis and visualised using a UV light and gel imaging system.

### 2.3 High‐throughput sequencing

A purified library was prepared according to Illumina library preparation protocols and transferred to an Illumina MiSeq platform for sequencing at Majorbio Co. Ltd. (Shanghai, China). Sequences with low‐quality reads were removed using the DADA2 denoising method in Qiime2 (Caporaso et al., 2010). The amplicon sequence variants (ASVs) from Illumina scale amplicon data without arbitrary dissimilarity thresholds were used for subsequent analyses (Callahan et al., 2017). Taxonomic classification of ASVs was completed using a local BLASTN (cut‐off E‐value 1e‐10) against the Ribosomal Database Project (RDP) database (Release 11) (Cole et al., 2013). All sequence data were deposited in GenBank under BioProject Accession. PRJNA1029771.

### 2.4 Calculation of community stability

*Vibrio* community stability was evaluated using the average variation degree (AVD), which was calculated using the degree of deviation from the mean of the normally distributed ASV relative abundance (Xun et al., 2021). Firstly, the degree of variation for each ASV was calculated using the following equation:

$$\left|a_{i}\right|=\frac{x_{i}-{\bar{x}}_{i}}{\delta_{i}}$$

where *a*_*i*_ is the variation degree for an ASV, *x*_*i*_ is the rarefied abundance of the ASV in one sample, ${\bar{x}}_{i}$ is the average rarefied abundance of the ASV in one sample group, and *δ*_*i*_ is the standard deviation of the rarefied abundances of the ASV in one sample group. Secondly, the AVD was calculated using the following equation:

$$\mathrm{AVD}=\frac{\sum_{i=1}^{n}\frac{x_{i}-{\bar{x}}_{i}}{\delta_{i}}}{k\times n}$$

where *k* is the number of samples in one sample group and *n* is the number of ASVs in each sample group.

### 2.5 Calculation of resource diversity

The resource diversity (RD) index was used to represent multidimensional resources in different habitats based on Liu et al. (2020). We chose TOC, total nitrogen (TN), and total phosphorus (TP), which represent the carbon, nitrogen, and phosphorus resources, respectively, to compute RD. We used the polygon radar chart method modified and improved by Hongliang et al. (2008) to calculate the differences in resources. The circumferential angle of each index varies with the total number of indices and index weight changes. The area (*S*) and perimeter (*L*) were expressed as:

$$S_{i}=\sum_{j}^{n}S_{j}=\sum_{j=1}^{n}\pi f_{j}r_{j}^{2}j=1,2\cdots ,n$$

$$L_{i}=\sum_{j}^{n}L_{j}=2\left|r_{\max}-r_{\min}\right|+\sum_{j=1}^{n}\pi f_{j}r_{j}^{2}j=1,2\cdots ,n$$

where *n* represents the number of indexes, *f*_*j*_ represents the weight of the *j*th index (each index was assigned the same weight in this study), and *r*_max_, *r*_min_, and *r*_*j*_ represent the maximum and minimum radii and radius of the *j*th index, respectively. Next, *v*_*i*1_ and *v*_*i*2_ were standardised to represent resource richness and evenness, respectively:

$${\mathrm{RR}}_{i}=S_{i}/\max S_{i}$$

$${\mathrm{RE}}_{i}=S_{i}/\left[\pi {\left(\frac{L_{i}}{2\pi }\right)}^{2}\right]=4\pi S_{i}/L_{i}^{2}$$

Based on *v*_*i*1_ and *v*_*i*2_, the RD index was calculated using the following:

$${\mathrm{RD}}_{i}=\sqrt{{\mathrm{RR}}_{i}{\mathrm{RE}}_{i}}$$

### 2.6 Calculation of phylogenetic diversity

We used phylogenetic species variability (PSV), phylogenetic species richness (PSR), and phylogenetic species evenness (PSE) to measure the phylogenetic diversity of the marine *Vibrio* community. The PSV measures the degree of phylogenetic relatedness of *Vibrio* species within a community (Guevara Andino et al., 2017) and was calculated using the following equation:

$$\mathrm{PSV}=\frac{\text{ntrC}-\sum C}{n\left(n-1\right)}$$

The *n* represents the number of species and *C* represents a covariance matrix, and *trC* is the sum of the diagonal elements of *C*, ∑ *C* indicates the sum of all elements in *C*.

PSR considers species richness and relatedness (Guevara Andino et al., 2017):

PSR=nPSV

PSE simultaneously considers the phylogenetic information and species evenness (Guevara Andino et al., 2017):

$$\mathrm{PSE}=\frac{\text{mdig}{\left(C\right)}^{\prime }M-M^{\prime }\mathrm{CM}}{m^{2}-{\bar{m}}_{i}m}$$

The PSE is derived from a community with a phylogeny given by *C* to a community with evolutionarily independent species with equal species abundances ($m_{i}={\bar{m}}_{i}$), where *m* is the total number of individuals and *m*_*i*_ is the number of individuals of species *i*. *M* is an *n* × 1 column vector containing values of *m*_*i*_ and diag gives an *n* × 1 column vector of the main diagonal of *C*. *M*′ is the transition of *M*.

### 2.7 Statistical analysis

We calculated the Richness, Shannon, Simpson, and Chao1 (Good, 1953; Kemp & Aller, 2004) indices, and using the Shannon index to represent the alpha diversity and Richness index to represent e the gamma diversity. We used the vegan package in R as follows: visualise the Bray Curtis dissimilarity of *Vibri*o communities using nonmetric multidimensional scaling (NMDS), and determine significant differences between communities through similarity analysis (ANOSIM). The impact of environmental factors on *Vibrio* communities was measured using the Mantel test. Correlations were calculated using the Spearman's rank method. Use R package “ggplot2” for linear regression analysis. Use the “plspm” package for partial least squares path model (PLS‐PM) analysis. As mentioned earlier, null model analysis was performed to classify the community assembly process (Stegen et al., 2013). Based on phylogenetic and taxonomic characteristics, the beta diversity metrics using the β‐nearest taxon index (βNTI) and Bray–Curtis‐based Raup‐Crick were generated for the evaluation of community assembly.

## 3 RESULTS

### 3.1 Composition and diversity of marine *Vibrio*

**FIGURE 1:**
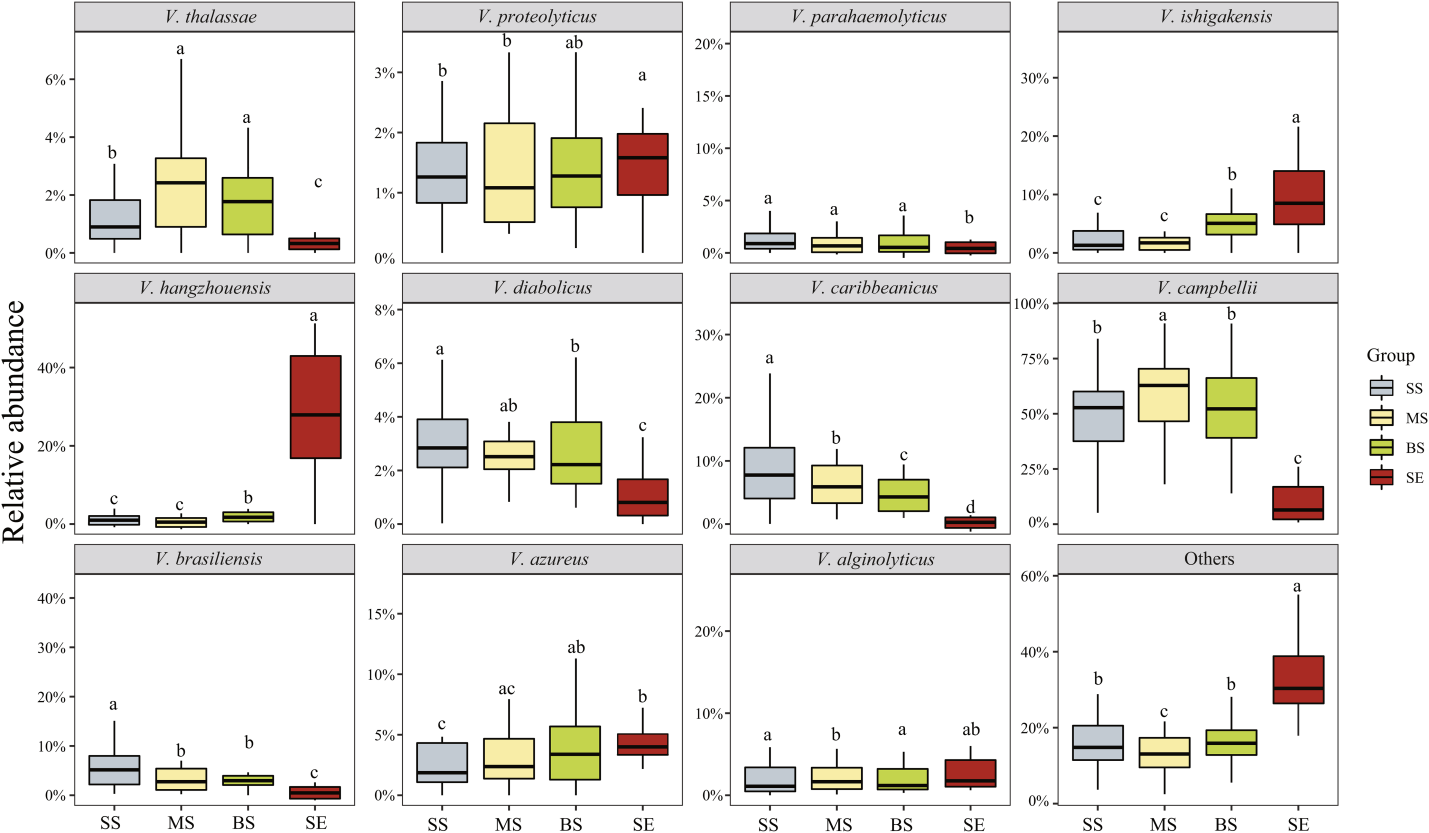
Relative abundances of 11 most abundant *Vibrio* species in different depth and habitats. BS, bottom layer of seawater; MS, middle layer of seawater; SE, sediment; SS, surface layer of seawater. Different letters indicate significant differences, while the same letters indicate non‐significant differences.

In total, 167,615 high‐quality sequences were obtained from all samples. The average number of detected sequences was 34,857 ± 1743 and the average ASV of each sample was 1252 ± 72. Good's coverage values were 98.07 ± 0.97, indicating that the vast majority of the *Vibrio* community was recovered. *V. campbellii* was the most abundant species in seawater, including SS, MS, BS samples (52.94%, 60.59%, 52.21%, respectively), followed by *V. carbbeanicus* (7.75%, 5.90%, 4.29%, respectively). *V. hangzhouensis* (27.91%) and *V. campbellii* (6.43%) were the most abundant species in SE samples (Figure 1). Besides, among the dominant *Vibrio* species, some common pathogenic strains, such as *V. parahaemolyticus*, *V. campbellii*, and *V. alginolyticus*, were detected in different layers of seawater and sediment samples. Notably, the proportions of *V. ishigakensis* and *V. azureus* increased vertically along the depth gradient from SS to SE. In contrast, the relative abundances of *V. diabolicus* and *V. caribbeanicus* gradually decreased (Figure 1).

**FIGURE 2:**
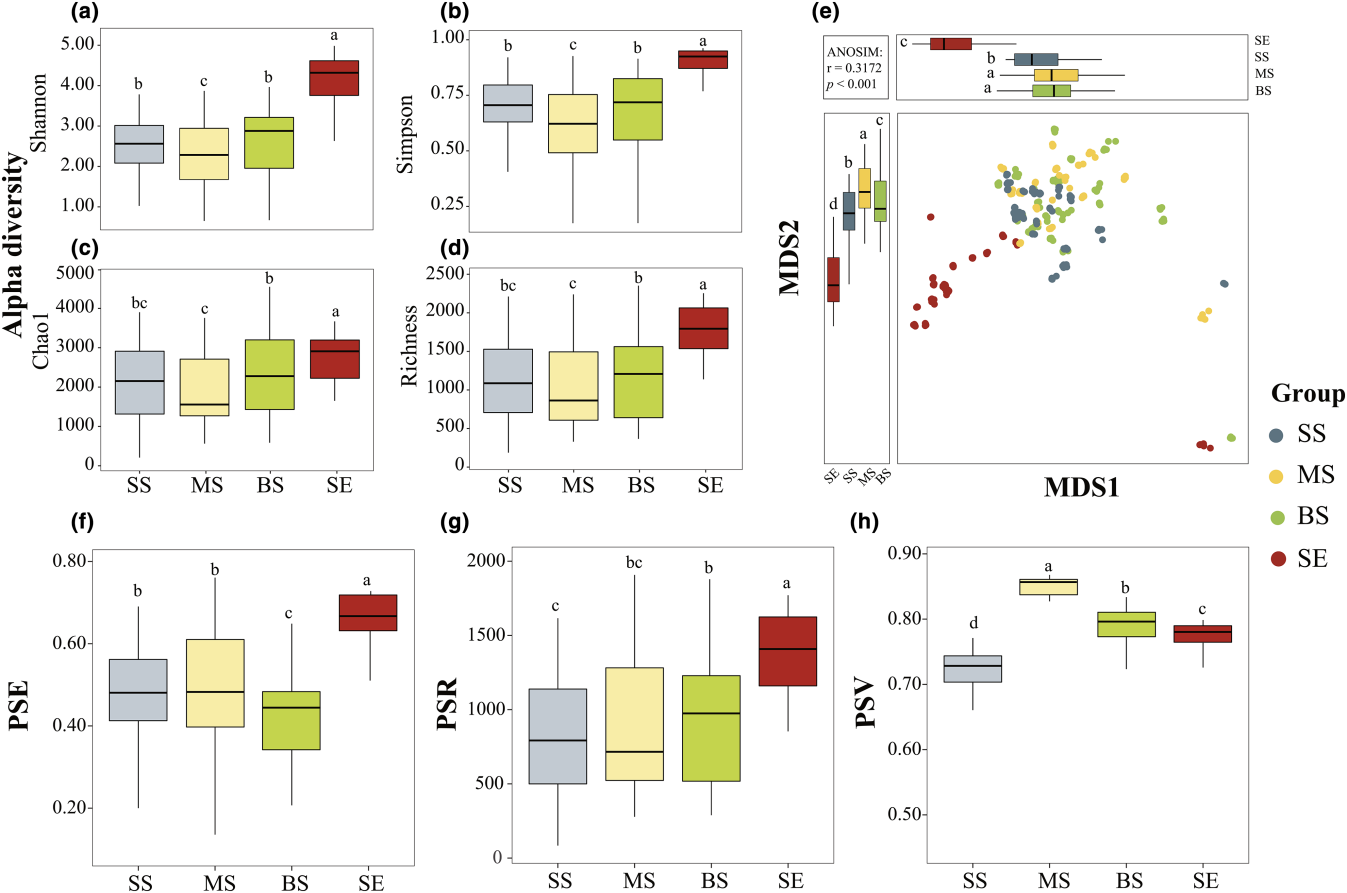
*Vibrio* alpha diversity and beta diversity in different depth and habitats. (a–d) alpha diversity (Shannon, Simpson, Chao 1, and Richness) presented by box plots; statistically significant differences (*p* < .05) are indicated by different letter for each group. (e) Beta diversity was calculated based on Bray–Curtis dissimilarity index and visualised by nonmetric multidimensional scaling (NMDS), which was performed to analyse similarity variation in the *Vibrio* community. Box plots represent differences among different depth and habitats. (f–h) Phylogenetic diversity presented by box plots. PSE, phylogenetic species evenness; PSR, phylogenetic species richness; PSV, phylogenetic species variability.

In this study, the Shannon and Richness indices were used to assess marine *Vibrio* alpha and gamma diversity, respectively. Alpha and gamma diversity were the highest in the SE group in comparison to the three other habitats groups, whereas the lowest alpha diversity observed in the MS group (Figure 2a–d). The NMDS plot based on the Bray‐Curtis distance revealed the variation between the marine *Vibrio* communities from all four habitats (Figure 2e).

SE samples have greater separation from SS, MS, and BS samples. The ANOSIM (*r* = .317, *p* < .001) test demonstrated significant differences in the beta diversity of the marine *Vibrio* community in different habitats. Phylogenetic diversity analysis revealed variations in PSV, PSR, and PSE across the different habitats. PSE and PSR values were highest in the SE sample (Figure 2f,g), whereas the PSV value was highest in the MS sample (Figure 2h). Additionally, the PSE value was lowest in the BS sample, PSR value was lowest in the MS sample, and PSV value was lowest in the SS sample (Figure 2f–h).

### 3.2 Assembly process of marine *Vibrio* community

**FIGURE 3:**
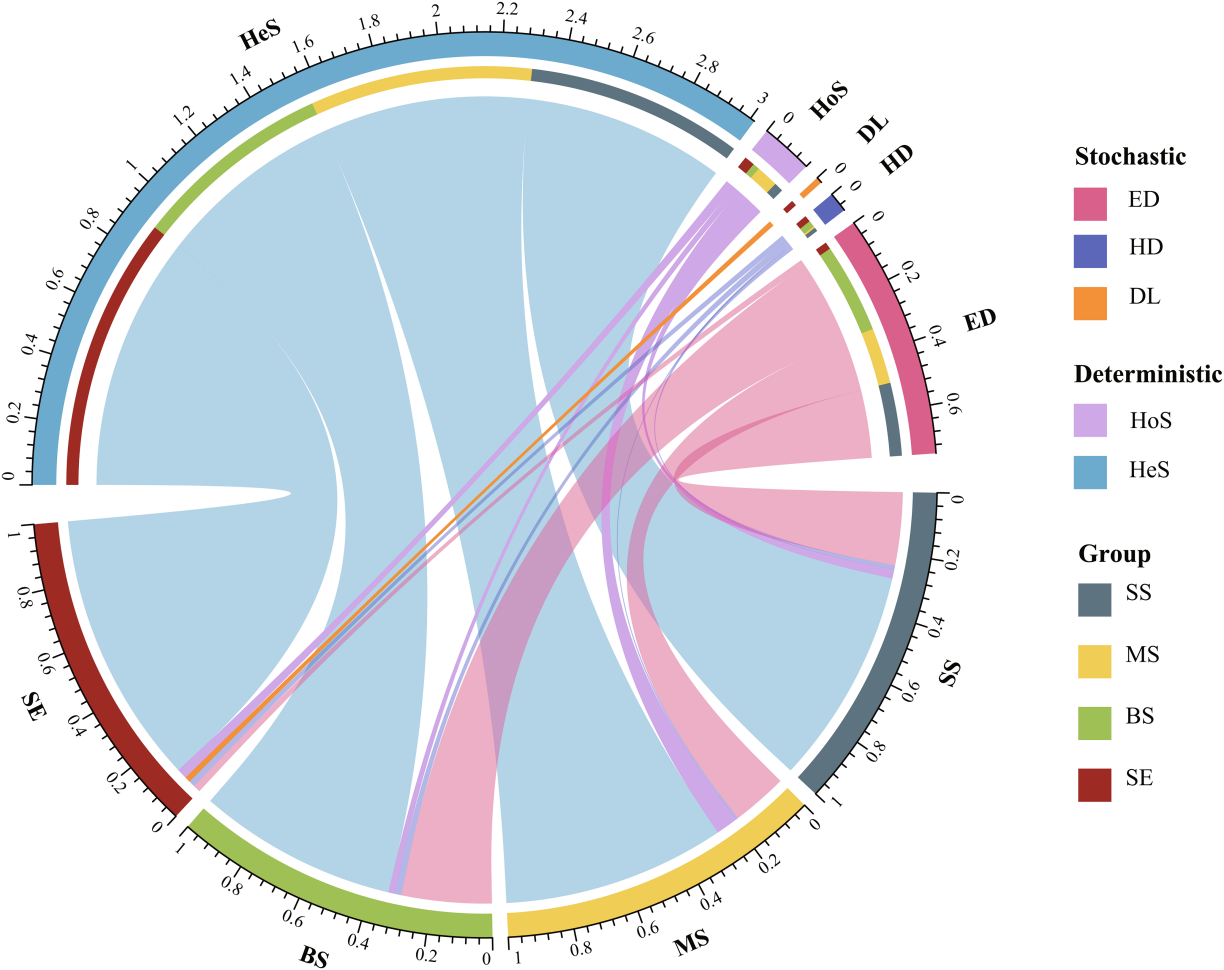
Analysis of *Vibrio* community assembly in different depth and habitats. DL, dispersal limitation; ED, ecological drift; HD, homogenising dispersal; HeS, heterogeneous selection; HoS, homogeneous selection.

βNTI was performed to evaluate the relative importance of stochastic and deterministic processes in shaping marine *Vibrio* community assembly in different habitats. |βNTI| ≥ 2 and |βNTI| ≤ 2 represent dominant deterministic processes and stochastic processes in shaping the marine *Vibrio* community, respectively. The proportions of βNTI values between >2 or <−2 were 71.62%, 80.92%, 68.16%, and 95.57% for the marine *Vibrio* community in SS, MS, BS, and SE, respectively (Figure 3). Deterministic rather than stochastic processes dominated the assembly of marine *Vibrio* communities in different habitats. Additionally, to explore the potential effects of deterministic or stochastic factors on marine *Vibrio* community structure, we assigned deterministic and stochastic processes to five specific ecological processes. In different layers of seawater (SS, MS, and BS samples) and SE samples, heterogeneous selection (HeS) dominated the marine *Vibrio* community, accounting for 71.57%, 73.56%, 65.96%, and 90.33%, respectively (Figure 3). For the SS, MS, and BS samples, the importance of ecological drift (ED) was second only to that of HeS, accounting for 24.10%, 18.46%, and 29.71%, respectively. The importance of homogeneous selection (HoS) was higher in SE samples (3.40%) than in ED samples (2.35%) (Figure 3). In general, deterministic processes made a more important contribution to the dynamics of marine *Vibrio* communities in different habitats, especially in SE, than stochastic processes.

### 3.3 Contribution of resource diversity to marine *Vibrio* diversity and community stability

**FIGURE 4:**
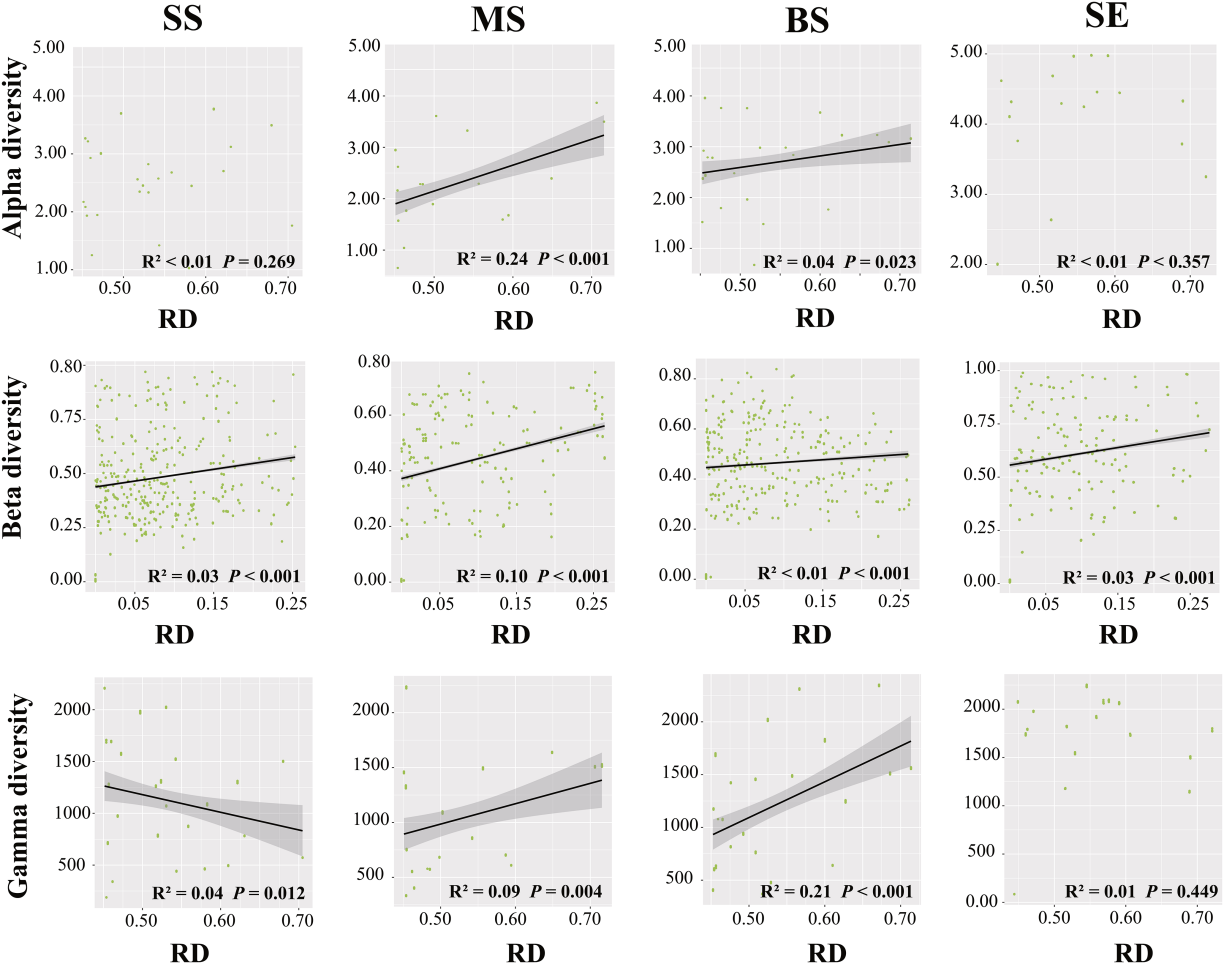
Linear regressions for RD associated with *Vibrio* alpha diversity, beta diversity, and gamma diversity.

**FIGURE 5:**
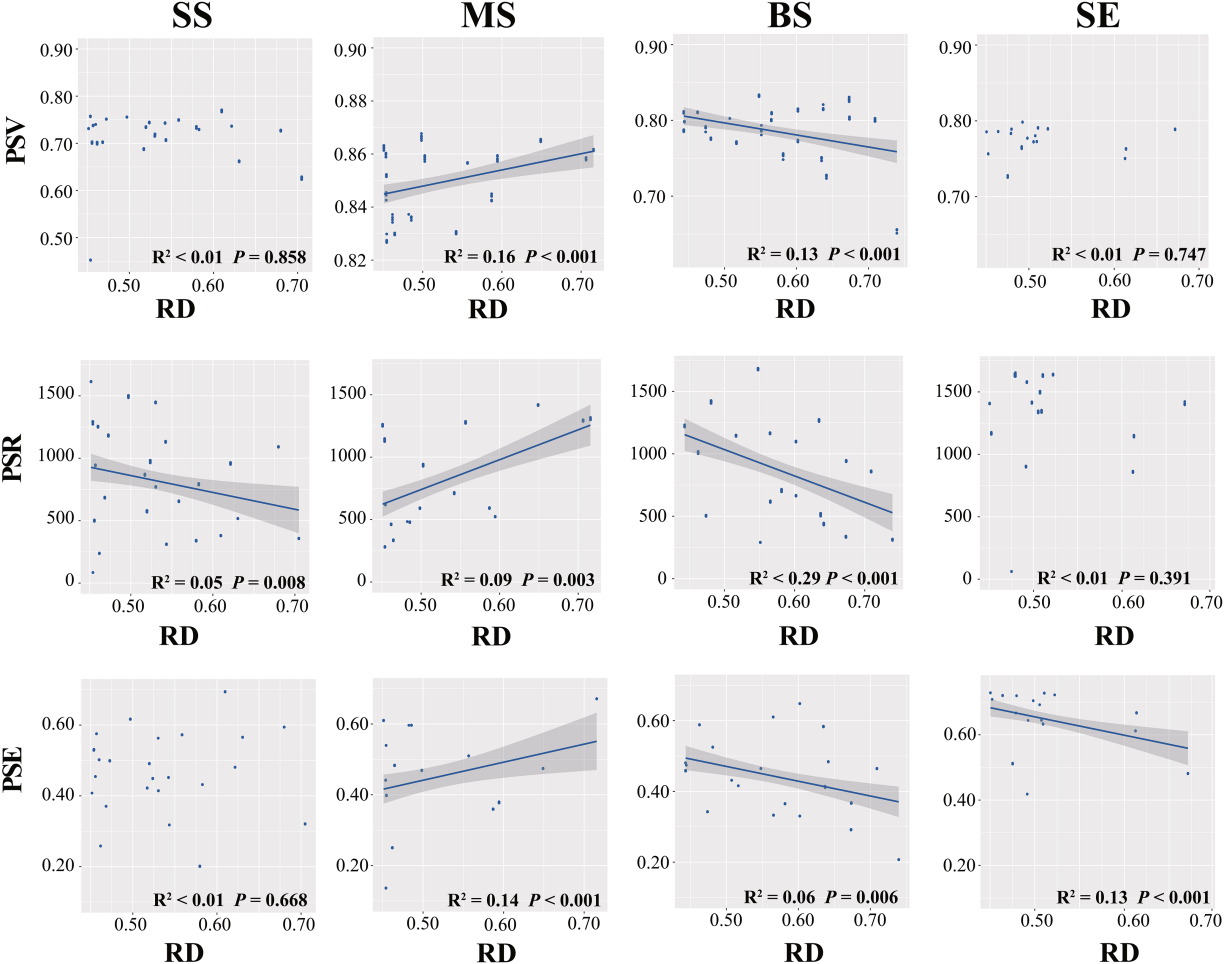
Linear regressions for phylogenetic diversity (PSE, PSR and PSV), associated with RD.

**FIGURE 6:**
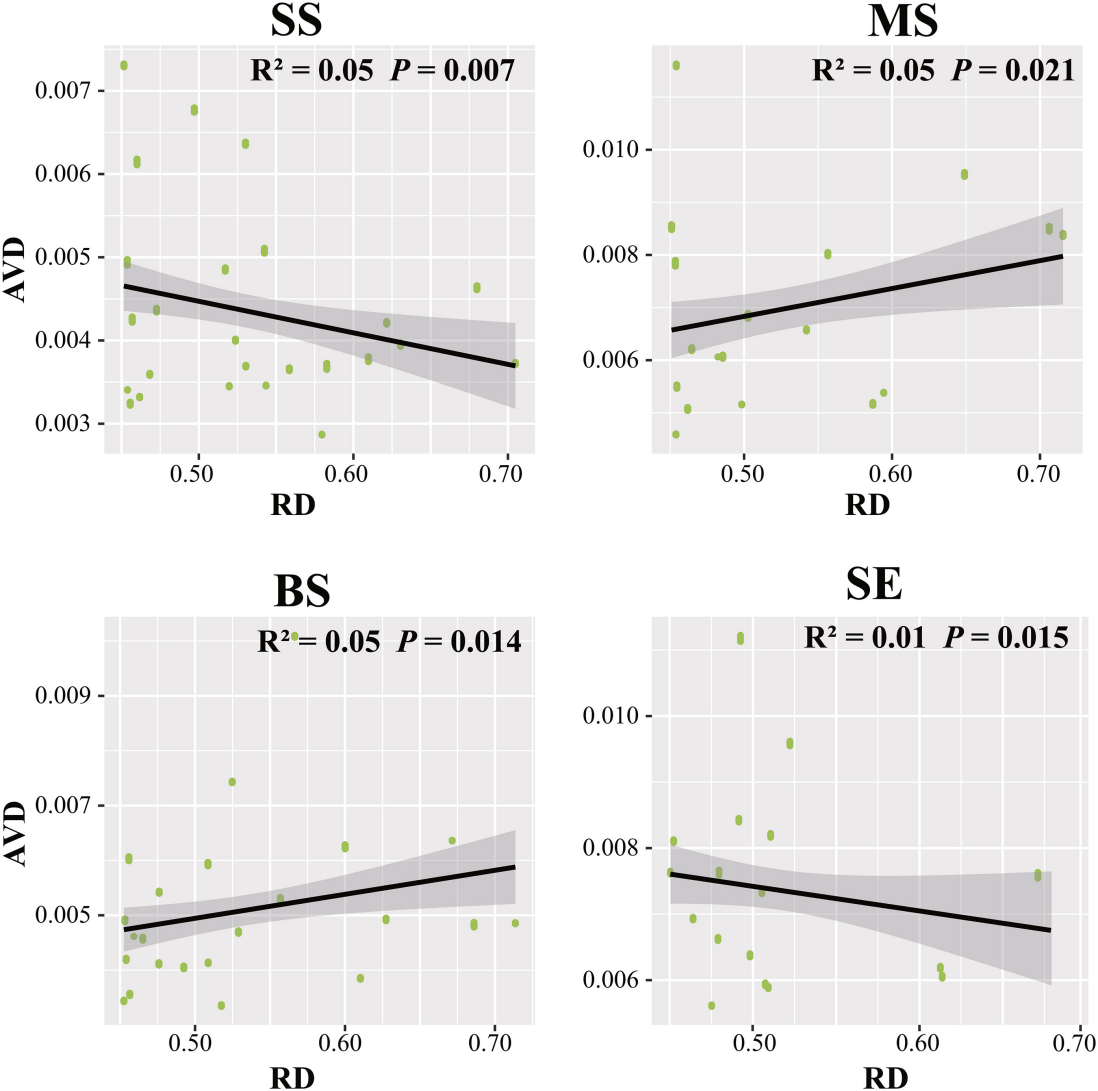
Linear regressions for RD associated with AVD in different habitats.

We explored the linear correlation between RD and taxonomic diversity. Alpha, beta, and gamma diversities were significantly positively correlated with RD in the MS and BS samples (*p* < .05) (Figure 4). In the SS samples, RD was significantly correlated with beta diversity (*r* ^2^ = .03, *p* < .001) and significantly negatively correlated with gamma diversity (*r* ^2^ = .04, *p* = .012) (Figure 4). Only beta diversity was significantly and positively correlated with RD in the sediment samples (*r* ^2^ = .03, *p* < .001) (Figure 4). For the impact of RD on and phylogenetic diversity, we found that the PSV, PSR, and PSE were significantly positively correlated with RD in the MS samples (*p* < .01), whereas the opposite trend was observed in the BS samples (Figure 5). In the SS samples, there was a significant positive correlation (*r* ^2^ = .05, *p* = .008) between PSR and RD and PSV, whereas only PSE was significantly negatively correlated with RD in the SE samples (*r* ^2^ = .13, *p* = .001) (Figure 5). Furthermore, we analysed the correlation between AVD and RD. In the SS and SE samples, AVD significantly decreased (*p* < .05) with increasing RD (Figure 6). Conversely, AVD was significantly and positively (*p* < .05) correlated with RD in BS and MS samples (Figure 6). Collectively, these results suggest that an increase in resource diversity has different effects on marine *Vibrio* diversity and community stability in different habitats.

**FIGURE 7:**
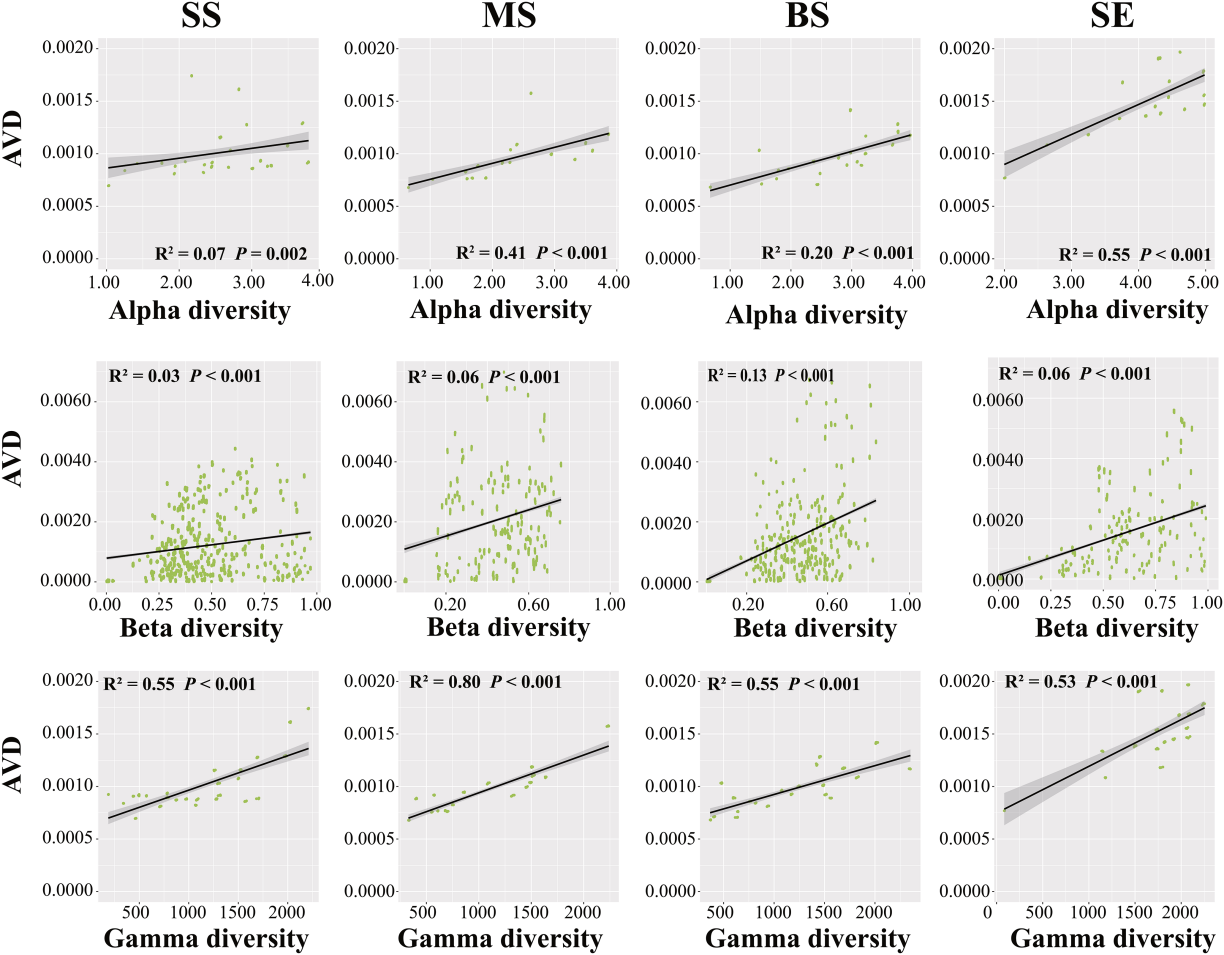
Linear regressions for AVD associated with marine *Vibrio* alpha diversity, beta diversity, and gamma diversity. *p*‐Values indicate significant differences.

**FIGURE 8:**
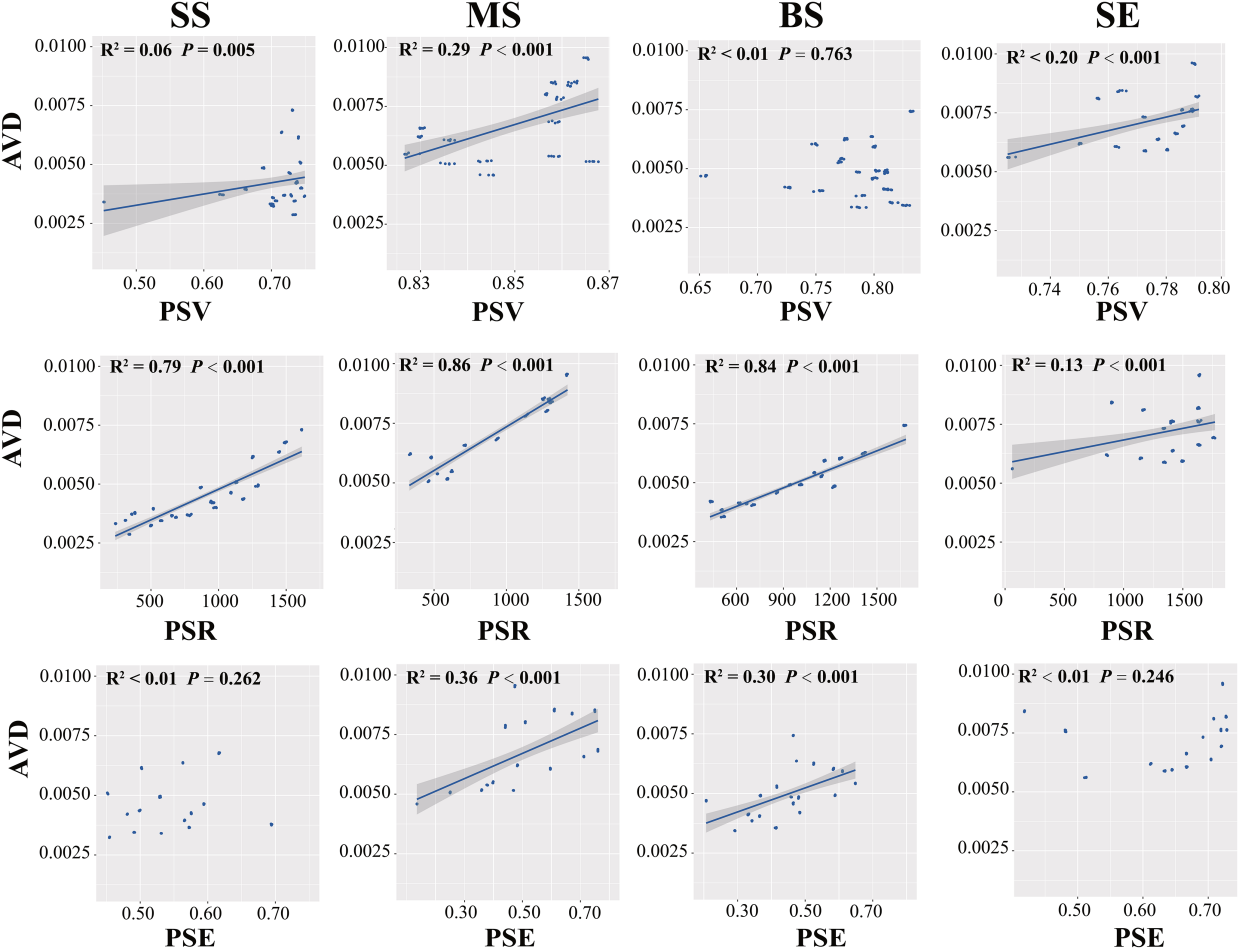
Linear regressions for phylogenetic diversity (PSE, PSR, and PSV) associated with AVD.

Linear regression analysis was used to investigate the correlation between the marine *Vibrio* diversity and community stability. In terms of the taxonomic diversity, AVD was significantly positively correlated with alpha, beta, and gamma diversity in all four habitats (*p* < .01) (Figure 7). The relationship between alpha diversity and AVD was strongest in SE samples (*r* ^2^ = .55, *p* < .001), whereas the beta diversity of BS samples had a stronger linear correlation with AVD than the other samples (*r* ^2^ = .13, *p* < .001), and gamma diversity showed the strongest positive correlation with AVD in the MS samples (Figure 7). Furthermore, Pearson's correlation analysis showed that in all habitats, beta and gamma diversities were significantly positively (*p* < .05) correlated with AVD (Table S1). Our results showed that increased taxonomic diversity weakened *Vibrio* community stability in different habitats. Moreover, for the phylogenetic diversity, the linear regression analysis demonstrated that PSV was positively correlated with AVD in the SS, MS, and SE samples (*p* < .01) (Figure 8). PSR was positively correlations with the AVD in all groups, and PSE was significantly positively correlated with AVD in the MS and BS samples (*p* < .001), but no significant correlation was observed in the SS and SE samples (*p* > .05) (Figure 8). Supplementally, Pearson's correlation analysis showed consistent results with the linear regression analysis, with significant positive correlations between AVD and PSV, PSE, and PSR in all groups (Table S1). These results suggest that, in all habitats, an increase in phylogenetic diversity leads to a decrease in the stability of the marine *Vibrio* community.

### 3.4 Environmental effects on diversity and community stability of marine *Vibrio*

Pearson's correlation and Mantel test analyses were performed to explore the key drivers of biodiversity and community stability in marine *Vibrio*. pH (*r* = .21, *p* < .05) and ${\mathrm{NH}}_{4}^{+}$‐N (*r* = −.22, *p* < .05) had the greatest impact on alpha and gamma diversity, respectively, in the MS samples, whereas DO (*r* = .27, *p* < .05) had the strongest effect on beta diversity (Tables S1 and S2). Furthermore, DIP (*r* = .25, *p* < .01), ${\mathrm{NO}}_{2}^{-}$‐N (*r* = .42, *p* < .001), and pH (*r* = .29, *p* < .001) exhibited the strongest correlations with PSV, PSR, and PSE (Table S1). In the MS samples, alpha, beta, and gamma diversity were significantly influenced by ${\mathrm{NH}}_{4}^{+}$‐N, DIN, TN, and TOC (*p* < .05) (Tables S1 and S2). Salinity (*r* = .44, *p* < .001) had the greatest impact on PSV, whereas DIN (*r* = −.41, *p* < .001) and TOC (*r* = .42, *p* < .001) had the strongest correlation with PSR and PSE, respectively (Table S1). For the BS samples, ${\mathrm{NO}}_{3}^{-}$‐N, pH, and TOC had a significant impact on alpha and gamma diversity (*p* < .05) (Table S1), whereas pH had the greatest impact on beta diversity (*r* = .25, *p* < .001) (Table S2). Additionally, ${\mathrm{NO}}_{3}^{-}$‐N (*r* = −.42, *p* < .001) had the greatest impact on PSV, TOC (*r* = .52, *p* < .001) had the strongest influence on PSR, and pH (*r* = −.49, *p* < .001) had the most significant effect on PSE (Table S1). In the SE group, DO, ${\mathrm{NO}}_{3}^{-}$‐N, ${\mathrm{NH}}_{4}^{+}$‐N, DIN, TN, DIP, and TP were significantly negatively correlated with alpha, beta, and gamma diversity (*p* < .05), whereas DO and DIP were significantly negatively correlated with PSV, PSR, and PSV (*p* < .001) (Tables S1 and S2). ${\mathrm{NO}}_{2}^{-}$‐N (*r* = .49, *p* < .001), DIN (*r* = −.48, *p* < .001), and ${\mathrm{NH}}_{4}^{+}$‐N (*r* = .25, *p* < .01) were the key driving factors of AVD in the SS, MS, and BS samples, respectively (Table S1). For the SE samples, salinity, pH, ${\mathrm{NH}}_{4}^{+}$‐N, and DIP were significantly correlated with AVD (*p* < .05) (Table S1). Collectively, these observations suggested that nutrients are important for the diversity and community stability of marine *Vibrio*.

Linear regression analysis was used to determine the potential impact of geographical distance on the taxonomic and phylogenetic diversity of marine *Vibrio*. Beta and gamma diversity significantly increased with increasing geographic distance (*p* < .05) (Figure S3). Alpha diversity was significantly positively correlated with geographic distance (*p* < .001) in the SS and SE samples, whereas a significant negative correlation was observed in the BS samples (*r* ^2^ < .01, *p* < .001), and there was no significant correlation in the MS samples (*p* > .05) (Figure S3). PSV, PSV, and PSR were significantly positively correlated with geographic distance in the SS and SE samples (*p* < .001). In the MS samples, PSV and PSR were significantly positively correlated with geographic distance (*p* < .001), whereas there was no significant correlation with PSE (*p* > .05) (Figure S4). Geographical distance was significantly positively correlated with PSR (*r* ^2^ < .01, *p* = .026) but significantly negatively correlated with PSE (*r* ^2^ < .01, *p* < .001), and there was no significant correlation with PSV (*p* > .05) in the BS samples (Figure S4).

**FIGURE 9:**
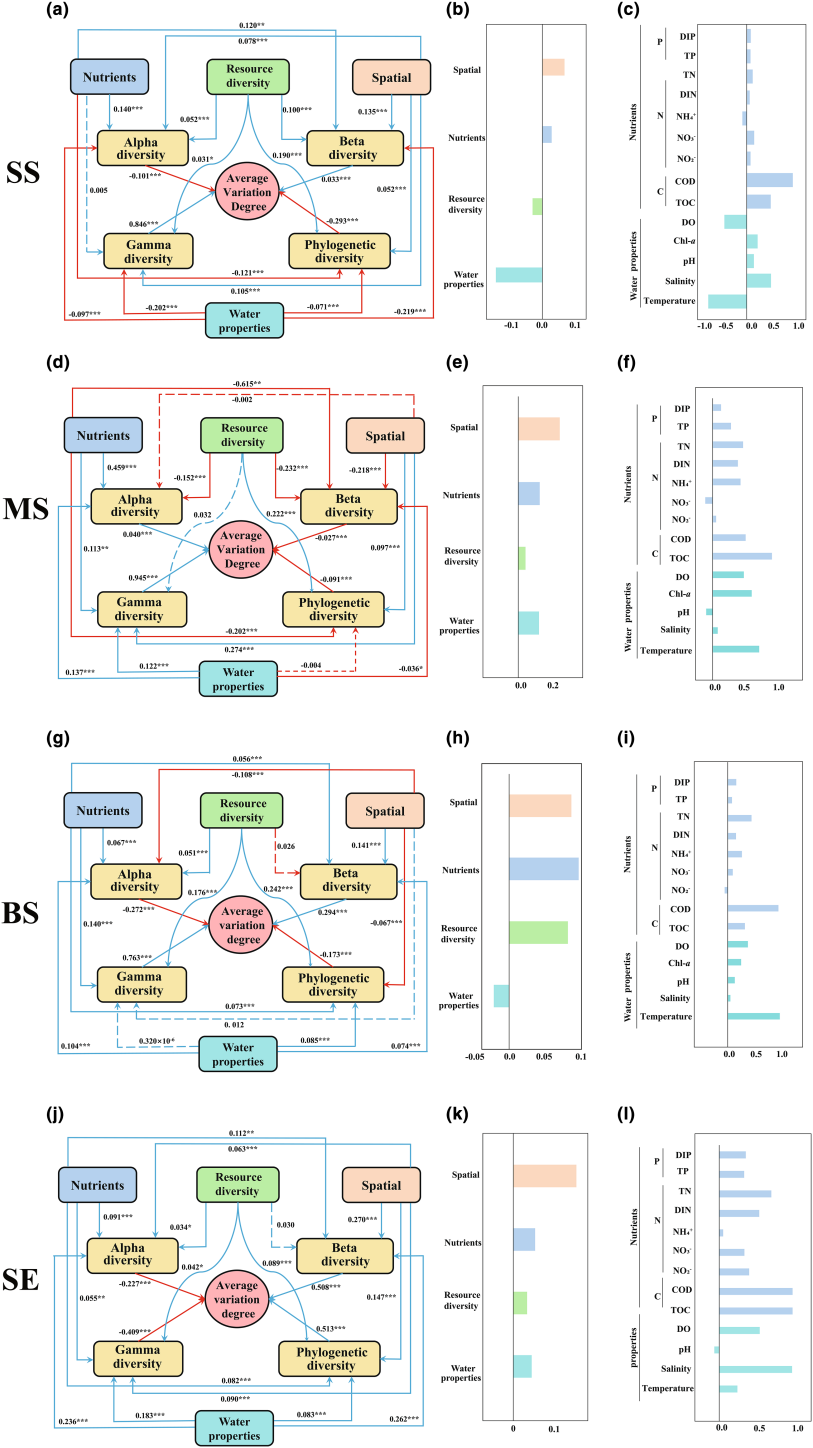
Path analysis representing the relationship among average variation degree, alpha diversity, beta diversity, gamma diversity, phylogenetic diversity, nutrients, resource diversity, spatial, and water properties in the subtropical sea. The red arrow represents a negative correlation, which means that an increase in the variation of the factors will reduce the diversity, and the blue arrow represents a positive correlation, which means that an increase in the variation of the factors will promote the diversity. The number is the *r* value; the asterisk indicates the *p* value: *p < .05; **p < .01; ***p < .001, and *Vibrio*.

PLS‐PM was constructed to analyse the relationship between AVD and taxonomic and phylogenetic diversity, as well as nutrients, water properties, RD, and spatial diversity, in different habitats. Nutrients had a significant positive (*p* < .001) effect on alpha diversity (Figure 9a). Water properties had the strongest and most significant (*p* < .001) effects on beta and gamma diversity in the SS samples (Figure 9a), and RD was identified as the most important factor influencing phylogenetic diversity (*p* < .001) (Figure 9a). In the case of MS samples, nutrients had the strongest significant (*p* < .001) effect on alpha and beta diversity (Figure 9d), *C* (COD and TOC) was the main contributing factor to nutrients (Figure 9c,f). Spatial variation and RD were the strongest factors that significantly (*p* < .001) influenced gamma diversity and phylogenetic diversity, respectively (Figure 9d). For the BS samples, spatial variation mainly affected alpha and beta diversity, and RD mainly affected gamma and phylogenetic diversity (*p* < .001) (Figure 9g). Additionally, in SE samples, water properties had the most significant effect on alpha and gamma diversity, whereas spatial variation had the strongest effects on beta and phylogenetic diversity (*p* < .001) (Figure 9j). Furthermore, PLS‐PM revealed that taxonomic and phylogenetic diversity jointly regulated marine *Vibrio* community stability. Specifically, gamma diversity had the strongest positive (*p* < .001) effect on AVD in the SS, MS, and BS samples (Figure 9a,g,j). Phylogenetic diversity mainly affected AVD in the SE samples (*p* < .001). Spatial variation had the strongest potential impact on AVD in the MS and SE groups (Figure 9e,k). RD had an indirect negative effect on AVD in the SS group (Figure 9b) and nutrients had the strongest indirect effect (Figure 9h). Overall, water properties, nutrients, RD, and spatial variation had the greatest effects on diversity. RD was the most important factor for phylogenetic diversity in all three seawater layers. The stability of the marine *Vibrio* community is regulated by taxonomic and phylogenetic diversity.

## 4 DISCUSSION

*Vibrio* occupies an important ecological niche in marine environments (Heidelberg et al., 2002; Zhou et al., 2021). Most studies on marine *Vibrio* ecology have focused on pathogenic groups or revealed their diversity and key environmental drivers in specific habitats (Amin et al., 2016; Jesser Kelsey et al., 2018; Mansergh & Zehr, 2014; Matteucci et al., 2015; Thompson, Randa, et al., 2004). However, environmental resources often exhibit enormous complexity, thus, quantifying multidimensional resource components can better elucidate the response mechanisms of marine *Vibrio* to environmental changes. To date, little is known about the effects of resource diversity on marine *Vibrio* diversity and community stability. In this study, we investigated the contribution of resource diversity to taxonomic and phylogenetic diversity, as well as community stability, in various habitats. Our results showed that resource diversity affects marine *Vibrio* diversity and community stability. Specifically, the influence of resource diversity on marine *Vibrio* community stability and diversity across distinct habitats, but the increase in taxonomic and phylogenetic diversity led to a reduction in the community stability of marine *Vibrio*.

### 4.1 Taxonomic and phylogenetic diversity of marine *Vibrio* varies in seawater and sediment samples

In recent years, many studies have been conducted on pathogenic *Vibrio* species that affect human and fishery health, especially in marginal seas severely affected by anthropogenic activities (Di et al., 2017; Froelich et al., 2017; Liang et al., 2019; Pruzzo et al., 2005; Vezzulli et al., 2009). For example, Di et al. (2017) found that *V. parahaemolyticus* and *V. alginolyticus* are ubiquitous in tidal water and mud year‐round along the southern coast of South Korea. Liang et al. (2019) reported that *V. campbellii* is dominant in the South Yellow Sea during summer. These findings suggest that the distribution of pathogenic *Vibrio* in marine environments is related to the impact of available resources derived from human activities such as aquaculture and pollution emissions. In our study, we identified three common pathogenic strains: *V. parahaemolyticus*, *V. campbellii*, and *V. alginolyticus*. The distribution of *V. parahaemolyticus* and *V. alginolyticus* in sediment and seawater was significantly lower than that of *V. campbellii*. Notedly, our results indicate that *V. hangzhouensis* is most abundant in the sediment of the Beibu Gulf, whereas *V. campbellii* exhibited the highest relative abundance in all three seawater habitats (Figure 1). We speculate that this phenomenon may be explained by the influence of the special habitat in the Beibu Gulf, which has led these species to exhibit diverse.

As expected, our findings suggest that both alpha and gamma diversity were highest in the sediment samples (Figure 2a–d). Similarly, (Wang et al., 2019; Wang, Liu, et al., 2022) found that in the marginal seas of China (including the Yellow Sea, East China Sea, and South China Sea), the diversity of marine *Vibrio* in sediments is generally higher than that in seawater. These findings corroborate previous evidence that sediments serve as a highly favourable ecological niche for marine *Vibrio* (Aravindraja et al., 2013; Vezzulli et al., 2009). Additionally, there is evidence that an increase in nutrient concentrations promotes an increase in marine *Vibrio* diversity (Li et al., 2020). Sediments harbour higher organic matter content than seawater (Hedges & Keil, 1995; Riley & Chester, 2013), which may result in the highest diversity of marine *Vibrio* observed in the sediment. The NMDS results illustrated that the marine *Vibrio* structure within the sediment exhibited a more pronounced divergence than that in other habitats. In contrast, the three seawater habitat samples partially overlapped (Figure 2e). This can be attributed to the distinct characteristics of seawater and sediment. Aquatic systems exhibit fluidity and interconnectedness (Chen et al., 2019; Zeng et al., 2019), and the continual vertical exchange of seawater amplifies the potential for taxonomic interchange among habitats. In contrast, sediments evolve over time through gradual accumulation, creating distinctive and stable conditions that facilitate the proliferation of marine *Vibrio*. Consequently, the discernible distinctions between seawater and sediment habitats are not surprising.

The *Vibrio* is highly diverse genetically (Travers et al., 2015). Here, we employed PSV, PSR, and PSE as comprehensive indicators to assess the phylogenetic diversity of marine *Vibrio*. The PSV primarily quantifies the degree to which species are phylogenetically related within a community. Compared to PSV, PSR incorporates species richness and phylogenetic information, whereas PSE integrates relative abundance while considering phylogenetic information and species consistency (Helmus et al., 2007). Our results demonstrated that PSE and PSR exhibited the highest values in the sediment samples, indicating that marine *Vibrio* species harbour greater species richness and more uniform distribution across phylogenetic branches in the sediment. This result can be attributed to the relatively stable environmental conditions in the sediment (Aller, 1994) which offer favourable conditions for the reproduction and survival of diverse marine *Vibrio* species, fostering their more equitable distribution across various phylogenetic lineages. Conversely, the highest degree of PSV was observed in the middle layer of seawater, suggesting that marine *Vibrio* populations in this habitat manifested higher variability in their phylogenetic development. The middle layer of seawater is exchanged with both the surface and bottom layers, resulting in stronger fluctuations in its physicochemical characteristics and complex conditions, potentially introducing a more complex and diverse environmental species pool. Consequently, these populations may experience greater selection pressure and adaptation requirements, leading to enhanced differentiation and diversity in their phylogenetic development.

### 4.2 Deterministic processes govern the phylogenetic community structure of marine *Vibrio* in different habitats

Pontarp et al. (2012) showed that habitat filtration shapes marine bacterial communities on a global scale. Pearson's correlation analysis revealed a strong correlation between marine *Vibrio* communities and various environmental factors in different habitats (Table S1). Therefore, we speculate that deterministic processes dominate the assembly process of marine *Vibrio* community. In this study, we used a null modelling‐based framework to detect the assembly process of the marine *Vibrio* community and revealed that deterministic processes were the main forces promoting the turnover of the marine *Vibrio* community in all four habitats, where heterogeneous selection was the predominant process. Heterogeneous selection is a deterministic concept that refers to different environmental selective forces that may drive a community towards greater dissimilarity (Dini‐Andreote et al., 2015; Vellend, 2010). The heterogeneous selection of the *Vibrio* community can be attributed to heterogeneous environmental factors that impose strong selective pressures (Li et al., 2021). Our research showed that βNTI was significantly correlated with most environmental factors, such as temperature, salinity, DIN, and DIP. Previous research has shown that abiotic factors such as temperature and nutrients are significantly related to the marine *Vibrio* community (Siboni et al., 2016; Takemura et al., 2014; Wright et al., 1996; Zhang et al., 2018). These factors have been proven to drive highly deterministic processes in various ecosystems (Guo et al., 2014; Lawrence & William, 2001; Zakem et al., 2018). Notably, our findings demonstrated the impact of stochastic processes mediated by ecological drift on *Vibrio* community in the surface, middle, and bottom layers of seawater. This effect stems from several causes. Firstly, certain *Vibrio* species perform similar functions, resulting in functional redundancy and niche overlap (Zhou & Ning, 2017), thereby increasing their potential for ecological drift. Secondly, the connectivity and mobility of seawater play a crucial role in promoting ecological drift (Chen et al., 2019; Zeng et al., 2019). Interestingly, our findings contradict a previous report of similar investigations in the Beibu gulf (Li et al., 2020). The reason for this contradiction may be attributed to the study of spatial scale differences. Shi et al. (2018) reported that deterministic patterns are more likely to be displayed on a large scale, whereas stochasticity tends to dominate on a local scale. Additionally, stochastic processes are more influential when there are few environmental variations or selection pressures (Shi et al., 2018). In this study, we sampled a wider range of spaces and diverse habitats, representing greater environmental differences and selection pressures. Consequently, deterministic processes dominated the construction of the marine *Vibrio* community. Furthermore, the regression analysis results revealed a significant positive correlation between marine *Vibrio* diversity and geographical distance (Figures S2 and S3), providing additional support for our conclusions.

### 4.3 Loss of taxonomic and phylogenetic diversity enhance community stability

The relationship between biodiversity and community stability has long been controversial in ecology (Hu et al., 2022; Huelsmann & Ackermann, 2022; Tilman et al., 2006). Owing to the intricate taxonomic and genetic diversity of microbial communities, characterising their interactions remains extremely challenging (Alivisatos et al., 2015). The prevailing consensus suggests that higher diversity contributes to enhanced community stability (Ives & Carpenter, 2007). However, contrasting views suggest that a higher microbial diversity may diminish community stability. For instance, Lehman and Tilman (Lehman & Tilman, 2000) researched a multi‐species competition model and their results showed that increase of biodiversity weakens community stability. Fischer et al. (2016) reported that increased gamma diversity results in decreased community stability. In our study, we observed a significant positive correlation between the average degree of variation and taxonomic and phylogenetic diversity, implying that a reduction in marine *Vibrio* diversity corresponds to an increase in community stability (Figures 7 and 8). Supplementally, Pearson's correlation analysis revealed that, in all habitats, beta and gamma diversity, as well as PSV, PSE, and PSR, were significantly positively (*p* < .05) correlated with AVD (Table S1). Previous studies have demonstrated that interaction strength is crucial for stability (Yodzis, 1981), and competition for resources among species may result in diminished community stability (Huelsmann & Ackermann, 2022). In general, the proportion of potential competitors participating in interspecific interference varies, and their interactions typically produce unstable effects (Yodzis, 1981). Additionally, the “hunger games” hypothesis suggests that cooperative interactions (e.g., cross‐feeding) are prevalent in bacterial communities, and a nutrient‐rich environment intensifies competition among species (Dai et al., 2022). Previous studies have demonstrated that marine *Vibrio* exhibit diverse metabolic abilities, including nitrogen fixation, phosphorus compound absorption, organic matter utilisation, and remineralisation (Thompson, Iida, & Swings, 2004; Zhang et al., 2018). By employing these distinct metabolic pathways and enzymatic systems, marine *Vibrio* species compete for essential resources, enabling survival and reproduction in complex and dynamic environments. Highly competitive species can exploit specific nutrient resources more efficiently, thereby gaining advantages in particular habitats. Overall, competition for nutrition among *Vibrio* species may result in stronger negative interactions, ultimately leading to a decrease in the community stability of marine *Vibrio*.

### 4.4 Resource diversity significantly affects marine *Vibrio* diversity and community stability

Previous studies have established that environmental factors exert a significant influence on marine *Vibrio* (Amin et al., 2016; Liang et al., 2019; Takemura et al., 2014). However, most of these investigations have been constrained to elucidating the individual contributions of distinct factors. In this study, we observed a substantial correlation between environmental factors and marine *Vibrio*. It is well known that the interplay between microbial communities and the environment is often intricate. Therefore, exclusive emphasis on the contribution of a single factor may not be sufficient to comprehensively unravel the intricate relationship between resources and the marine *Vibrio* community (Wang, Du, et al., 2022).

To comprehensively quantify multidimensional resources, we employed the RD index, which considers both the average abundance of resources and their balance (Wang, Du, et al., 2022), to evaluate resource diversity across various marine habitats, and revealed its impact on the diversity and community stability of marine *Vibrio*. PLS‐PM showed that the effects of RD on alpha, beta, and gamma diversity exhibited heterogeneous patterns across the four studied habitats (Figures 8 and 9a,d,g,j). Specifically, RD had a negative effect on alpha diversity only in the middle layer of seawater (Figure 9d), whereas it had a positive effect in other habitats. Furthermore, RD was positively correlated with beta diversity in the surface and middle layers of seawater (Figure 9a,d). However, no significant correlation emerged between RD and gamma diversity, except in the middle seawater layer (Figure 9d). These findings underscore the intricate relationship between diversity metrics and resource diversity, revealing inherent inconsistency (Muscarella et al., 2019). At the vertical level, these inconsistencies may have been caused by habitat differences. Temperature and salinity are the most common key factors affecting marine *Vibrio*, whereas other parameters vary according to the habitat. Geographical distance may be another pivotal factor. Previous studies have shown that marine *Vibrio* in seawater and sediments are related to geographical distance (Wang et al., 2019; Wang, Liu, et al., 2022). Our results showed that alpha, beta, and gamma diversities were significantly correlated with geographic distance in almost all habitats (Figure S3). These differences may be attributed to variations in competitive ability (Leibold, 1999) and shared limitations across species (Stevens & Carson, 2002). It is worth noting that RD exerted a positive effect on phylogenetic diversity in all four habitats (Figure 9a,d,g,j). Theoretically, resource enrichment can foster increased diversity, thereby supporting more intricate microbial communities (Smith, 2007; Worm et al., 2002) and, ultimately, promoting gene flow and maintaining genetic diversity within species (Beger et al., 2014; Fu et al., 2016). Moreover, when resources in the ecosystem are abundant and diverse, species may be more likely to cope with environmental pressures, reducing the risk of individual genetic damage and maintaining phylogenetic diversity.

Regression analysis showed that RD and AVD exhibited different patterns in different habitats, with a negative correlation in the surface layer of seawater and sediment and a positive correlation in the middle and bottom layers. This indicates that an increase in resource diversity in the surface water and sediments enhances the marine *Vibrio* community stability. However, the opposite trend was observed in the middle and bottom layers. Additionally, PLS‐PM indicated that RD indirectly affects community stability through diversity. Previous studies have shown that there are usually more biologically available resources (such as light energy and organic carbon) in surface water and sediment (Garel et al., 2019; Hedges & Keil, 1995; Riley & Chester, 2013; Skoog & Benner, 1997) and that the diversity of these resources may support the survival and reproduction of different biological species, thereby promoting community stability. Middle and bottom water resources are relatively scarce and experience greater changes in temperature and pressure (Garel et al., 2019), which may have different degrees of impact on species, leading to a decline in community stability.

## 5 CONCLUSION

In this study, the spatial distribution of marine *Vibrio* in different habitats was determined using PCR and high‐throughput sequencing. *V. hangzhouensis* was most abundant in sediment in the Beibu Gulf, whereas *V. campbellii* was the dominant species in all layers of seawater. Sediments exhibited higher alpha and gamma diversity, and nutrients played a crucial role in the diversity and community stability of marine *Vibrio*. We further revealed that marine *Vibrio* community assembly was dominated by deterministic processes. Moreover, marine *Vibrio* community stability was mainly influenced by ${\mathrm{NO}}_{2}^{-}$‐N, DIN, and ${\mathrm{NH}}_{4}^{+}$‐N in seawater, whereas pH was the main factor in sediment. Resource diversity, water properties, nutrients, and geographical distance had significant effects on marine *Vibrio* diversity and community stability. Overall, our results show that the influence of resource diversity on marine *Vibrio* diversity and community stability varies with habitat, however, the loss of taxonomic and phylogenetic diversity simultaneously enhanced community stability. These findings contribute to the understanding of the contribution of resource diversity to heterotrophic bacterial diversity and community stability.

## AUTHOR CONTRIBUTIONS

**Xinyi Qin:** Conceptualization (equal); methodology (equal); supervision (equal); writing – review and editing (equal). **Qinghua Hou:** Conceptualization (equal); data curation (equal); formal analysis (equal); investigation (equal); methodology (equal); visualization (equal); writing – original draft (equal); writing – review and editing (equal). **Huaxian Zhao:** Writing – review and editing (equal). **Pengbin Wang:** Writing – review and editing (equal). **Shu Yang:** Investigation (equal); methodology (equal); supervision (equal); visualization (equal). **Nengjian Liao:** Writing – review and editing (equal). **Jiongqing Huang:** Investigation (equal); supervision (equal). **Xiaoli Li:** Writing – review and editing (equal). **Qing He:** Formal analysis (equal); investigation (equal); supervision (equal). **Rajapakshalage Thashikala Nethmini:** Formal analysis (equal); investigation (equal); supervision (equal). **Gonglingxia Jiang:** Formal analysis (equal); visualization (equal). **Shiying He:** Supervision (equal); writing – review and editing (equal). **Qingxiang Chen:** Supervision (equal); writing – review and editing (equal). **Ke Dong:** Writing – review and editing (equal). **Nan Li:** Conceptualization (equal); data curation (equal); formal analysis (equal); investigation (equal); methodology (equal); visualization (equal); writing – original draft (equal); writing – review and editing (equal).

## CONFLICT OF INTEREST STATEMENT

The authors declare that they have no known competing financial interests or personal relationships that could have appeared to influence the work reported in this paper.

## CONSENT FOR PUBLICATION

All authors consent for publication.

## Supporting information


Figures S1–S4.

Tables S1 and S2.

## Data Availability

The datasets presented in this study are found in NCBI SRA as BioProject PRJNA1029771, the sediment samples access numbers ranging from SAMN38054830 to SAMN38054914 and seawater samples ranging from SAMN38054423 to SAMN38054742.

## References

[ece311234-bib-0001] Alivisatos, A. P. , Blaser, M. J. , Brodie, E. L. , Chun, M. , Dangl, J. L. , Donohue, T. J. , & Consortium, U. M. I. (2015). A unified initiative to harness earth's microbiomes. Science, 350(6260), 507–508. 10.1126/science.aac8480 26511287

[ece311234-bib-0002] Aller, R. C. (1994). Bioturbation and remineralization of sedimentary organic matter: Effects of redox oscillation. Chemical Geology, 114(3), 331–345. 10.1016/0009-2541(94)90062-0

[ece311234-bib-0003] American Public Health Association . (1926). Standard methods for the examination of water and wastewater (Vol. 6). American Public Health Association.

[ece311234-bib-0004] Amin, A. K. M. R. , Feng, G. , Al‐saari, N. , Meirelles, P. M. , Yamazaki, Y. , Mino, S. , & Sawabe, T. (2016). The first temporal and spatial assessment of vibrio diversity of the surrounding seawater of coral reefs in Ishigaki, Japan. Frontiers in Microbiology, 7, 1185. 10.3389/fmicb.2016.01185 27551278 PMC4976104

[ece311234-bib-0005] Aravindraja, C. , Viszwapriya, D. , & Karutha Pandian, S. (2013). Ultradeep 16S rRNA sequencing analysis of geographically similar but diverse unexplored marine samples reveal varied bacterial community composition. PLoS One, 8(10), e76724. 10.1371/journal.pone.0076724 24167548 PMC3805540

[ece311234-bib-0006] Beger, M. , Selkoe, K. A. , Treml, E. , Barber, P. H. , von der Heyden, S. , Crandall, E. D. , & Riginos, C. (2014). Evolving coral reef conservation with genetic information. Bulletin of Marine Science, 90(1), 159–185. 10.5343/bms.2012.1106

[ece311234-bib-0007] Cadotte, M. W. , Dinnage, R. , & Tilman, D. (2012). Phylogenetic diversity promotes ecosystem stability. Ecology, 93, S223–S233. 10.1890/11-0426.1

[ece311234-bib-0008] Callahan, B. J. , McMurdie, P. J. , & Holmes, S. P. (2017). Exact sequence variants should replace operational taxonomic units in marker‐gene data analysis. The ISME Journal, 11(12), 2639–2643. 10.1038/ismej.2017.119 28731476 PMC5702726

[ece311234-bib-0009] Caporaso, J. G. , Kuczynski, J. , Stombaugh, J. , Bittinger, K. , Bushman, F. D. , Costello, E. K. , & Gordon, J. I. (2010). QIIME allows analysis of high‐throughput community sequencing data. Nature Methods, 7(5), 335–336. 10.1038/nmeth.f.303 20383131 PMC3156573

[ece311234-bib-0010] Chase, J. M. , Kraft, N. J. B. , Smith, K. G. , Vellend, M. , & Inouye, B. D. (2011). Using null models to disentangle variation in community dissimilarity from variation in α‐diversity. Ecosphere, 2(2), art24. 10.1890/ES10-00117.1

[ece311234-bib-0011] Chen, W. , Ren, K. , Isabwe, A. , Chen, H. , Liu, M. , & Yang, J. (2019). Stochastic processes shape microeukaryotic community assembly in a subtropical river across wet and dry seasons. Microbiome, 7, 1–16. 10.1186/s40168-019-0749-8 31640783 PMC6806580

[ece311234-bib-0012] Chen, X. , Zhao, H. , Jiang, G. , Tang, J. , Xu, Q. , Huang, L. , & Li, N. (2020). Responses of free‐living *Vibrio* community to seasonal environmental variation in a subtropical Inland Bay. Frontiers in Microbiology, 11, 610974. 10.3389/fmicb.2020.610974 33381102 PMC7767907

[ece311234-bib-0013] Chen, Z. , Xu, S. , Qiu, Y. , Lin, Z. , & Jia, X. (2009). Modeling the effects of fishery management and marine protected areas on the Beibu gulf using spatial ecosystem simulation. Fisheries Research, 100(3), 222–229. 10.1016/j.fishres.2009.08.001

[ece311234-bib-0014] Chiang, S.‐R. , & Chuang, Y.‐C. (2003). *Vibrio vulnificus* infection: Clinical manifestations, pathogenesis, and antimicrobial therapy. Journal of Microbiology, Immunology, and Infection, 36(2), 81–88. 12886957

[ece311234-bib-0015] Cole, J. R. , Wang, Q. , Fish, J. A. , Chai, B. , McGarrell, D. M. , Sun, Y. , & Tiedje, J. M. (2013). Ribosomal database project: Data and tools for high throughput rRNA analysis. Nucleic Acids Research, 42(D1), D633–D642. 10.1093/nar/gkt1244 24288368 PMC3965039

[ece311234-bib-0016] Colwell, R. R. , & Spira, W. M. (1992). The ecology of *Vibrio cholerae* . In D. Barua & W. B. Greenough (Eds.), Cholera (pp. 107–127). Springer.

[ece311234-bib-0017] Craven, D. , Eisenhauer, N. , Pearse, W. D. , Hautier, Y. , Isbell, F. , Roscher, C. , Bahn, M. , Beierkuhnlein, C. , Bönisch, G. , Buchmann, N. , Byun, C. , Catford, J. A. , Cerabolini, B. E. L. , Cornelissen, J. H. C. , Craine, J. M. , De Luca, E. , Ebeling, A. , Griffin, J. N. , Hector, A. , … Manning, P. (2018). Multiple facets of biodiversity drive the diversity–stability relationship. Nature Ecology & Evolution, 2(10), 1579–1587. 10.1038/s41559-018-0647-7 30150740

[ece311234-bib-0018] Dai, T. , Wen, D. , Bates, C. T. , Wu, L. , Guo, X. , Liu, S. , & Yang, Y. (2022). Nutrient supply controls the linkage between species abundance and ecological interactions in marine bacterial communities. Nature Communications, 13(1), 175. 10.1038/s41467-021-27857-6 PMC8748817 35013303

[ece311234-bib-0019] Di, D. Y. , Lee, A. , Jang, J. , Han, D. , & Hur, H.‐G. (2017). Season‐specific occurrence of potentially pathogenic *Vibrio* spp. on the southern coast of South Korea. Applied and Environmental Microbiology, 83(3), e02680‐16. 10.1128/AEM.02680-16 27836844 PMC5244290

[ece311234-bib-0020] Dini‐Andreote, F. , Stegen, J. C. , van Elsas, J. D. , & Salles, J. F. (2015). Disentangling mechanisms that mediate the balance between stochastic and deterministic processes in microbial succession. Proceedings of the National Academy of Sciences of the United States of America, 112(11), E1326–E1332. 10.1073/pnas.1414261112 25733885 PMC4371938

[ece311234-bib-0021] Donohue, I. , Petchey, O. L. , Montoya, J. M. , Jackson, A. L. , McNally, L. , Viana, M. , Healy, K. , Lurgi, M. , O'Connor, N. E. , & Emmerson, M. C. (2013). On the dimensionality of ecological stability. Ecology Letters, 16(4), 421–429. 10.1111/ele.12086 23419041

[ece311234-bib-0022] Erkus, O. , de Jager, V. C. L. , Spus, M. , van Alen‐Boerrigter, I. J. , van Rijswijck, I. M. H. , Hazelwood, L. , & Smid, E. J. (2013). Multifactorial diversity sustains microbial community stability. The ISME Journal, 7(11), 2126–2136. 10.1038/ismej.2013.108 23823494 PMC3806261

[ece311234-bib-0023] Farmer, J., III , Michael Janda, J. , Brenner, F. W. , Cameron, D. N. , & Birkhead, K. M. (2015). Vibrio. In W. B. Whitman (Ed.), Bergey's manual of systematics of archaea and bacteria (pp. 1–79). John Wiley & Sons, Inc.

[ece311234-bib-0024] Fischer, F. M. , Wright, A. J. , Eisenhauer, N. , Ebeling, A. , Roscher, C. , Wagg, C. , & Pillar, V. D. (2016). Plant species richness and functional traits affect community stability after a flood event. Philosophical Transactions of the Royal Society of London. Series B, Biological Sciences, 371(1694), 20150276. 10.1098/rstb.2015.0276 27114578 PMC4843697

[ece311234-bib-0025] Froelich, B. , Phippen, B. , Fowler, P. , Noble, R. , & Oliver, J. (2017). Differences in abundances of total *Vibrio* spp., *V. vulnificus*, and *V. parahaemolyticus* in clams and oysters in North Carolina. Applied and Environmental Microbiology, 83(2), e02265‐16. 10.1128/AEM.02265-16 27793822 PMC5203622

[ece311234-bib-0026] Fu, P. C. , Gao, Q. B. , Zhang, F. Q. , Xing, R. , Wang, J. L. , Liu, H. R. , & Chen, S. L. (2016). Gene flow results in high genetic similarity between Sibiraea (Rosaceae) species in the Qinghai‐Tibetan plateau. Frontiers in Plant Science, 7, 1596. 10.3389/fpls.2016.01596 27826314 PMC5078775

[ece311234-bib-0027] Garel, M. , Bonin, P. , Martini, S. , Guasco, S. , Roumagnac, M. , Bhairy, N. , & Tamburini, C. (2019). Pressure‐retaining sampler and high‐pressure systems to study Deep‐Sea microbes under in situ conditions. Frontiers in Microbiology, 10, 453. 10.3389/fmicb.2019.00453 31024462 PMC6465632

[ece311234-bib-0028] Good, I. J. (1953). The population frequencies of species and the estimation of population parameters. Biometrika, 40(3–4), 237–264. 10.1093/biomet/40.3-4.237

[ece311234-bib-0029] Guevara Andino, J. E. , Pitman, N. C. A. , ter Steege, H. , Mogollón, H. , Ceron, C. , Palacios, W. , & Fine, P. V. A. (2017). Incorporating phylogenetic information for the definition of floristic districts in hyperdiverse Amazon forests: Implications for conservation. Ecology and Evolution, 7(22), 9639–9650. 10.1002/ece3.3481 29187996 PMC5696432

[ece311234-bib-0030] Guin, S. , Saravanan, M. , Chowdhury, G. , Pazhani, G. P. , Ramamurthy, T. , & Das, S. C. (2019). Pathogenic *Vibrio parahaemolyticus* indiarrhoeal patients, fish and aquatic environments and their potential for inter‐source transmission. Heliyon, 5(5), e01743. 10.1016/j.heliyon.2019.e01743 31193375 PMC6526242

[ece311234-bib-0031] Guo, S. , Feng, Y. , Wang, L. , Dai, M. , Liu, Z. , Bai, Y. , & Sun, J. (2014). Seasonal variation in the phytoplankton community of a continental‐shelf sea: The East China Sea. Marine Ecology Progress Series, 516, 103–126. 10.3354/meps10952

[ece311234-bib-0032] Hedges, J. I. , & Keil, R. G. (1995). Sedimentary organic matter preservation: An assessment and speculative synthesis. Marine Chemistry, 49(2–3), 81–115. 10.1016/0304-4203(95)00008-F

[ece311234-bib-0033] Heidelberg, J. F. , Heidelberg, K. B. , & Colwell, R. R. (2002). Bacteria of the gamma‐subclass Proteobacteria associated with zooplankton in Chesapeake Bay. Applied and Environmental Microbiology, 68(11), 5498–5507. 10.1128/aem.68.11.5498-5507.2002 12406743 PMC129896

[ece311234-bib-0034] Helmus, M. R. , Bland, T. J. , Williams, C. K. , & Ives, A. R. (2007). Phylogenetic measures of biodiversity. The American Naturalist, 169(3), E68–E83. 10.1086/511334 17230400

[ece311234-bib-0035] Hongliang, L. , Anxin, L. , Bin, Z. , Tiefu, Z. , & Xin, Z. (2008). A fuzzy comprehensive evaluation method of maintenance quality based on improved radar chart . Paper presented at the 2008 ISECS International Colloquium on Computing, Communication, Control, and Management.

[ece311234-bib-0036] Hou, X. M. , Xu, R. F. , Gu, Y. C. , Wang, C. Y. , & Shao, C. L. (2015). Biological and chemical diversity of coral‐derived microorganisms. Current Medicinal Chemistry, 22(32), 3707–3762. 10.2174/0929867322666151006093755 26438250

[ece311234-bib-0037] Hu, J. , Amor, D. R. , Barbier, M. , Bunin, G. , & Gore, J. (2022). Emergent phases of ecological diversity and dynamics mapped in microcosms. Science, 378(6615), 85–89. 10.1126/science.abm7841 36201585

[ece311234-bib-0038] Huelsmann, M. , & Ackermann, M. (2022). Community instability in the microbial world. Science, 378(6615), 29–30. 10.1126/science.ade2516 36201571

[ece311234-bib-0039] Ives, A. R. , & Carpenter, S. R. (2007). Stability and diversity of ecosystems. Science, 317(5834), 58–62. 10.1126/science.1133258 17615333

[ece311234-bib-0040] Jesser Kelsey, J. , Noble Rachel, T. , & Elkins Christopher, A. (2018). Vibrio ecology in the Neuse River estuary, North Carolina, characterized by next‐generation amplicon sequencing of the gene encoding heat shock protein 60 (hsp60). Applied and Environmental Microbiology, 84(13), e00333‐18. 10.1128/AEM.00333-18 29678912 PMC6007114

[ece311234-bib-0041] Jiao, S. , Wang, J. , Wei, G. , Chen, W. , & Lu, Y. (2019). Dominant role of abundant rather than rare bacterial taxa in maintaining agro‐soil microbiomes under environmental disturbances. Chemosphere, 235, 248–259. 10.1016/j.chemosphere.2019.06.174 31260865

[ece311234-bib-0042] Kemp, P. F. , & Aller, J. Y. (2004). Bacterial diversity in aquatic and other environments: What 16S rDNA libraries can tell us. FEMS Microbiology Ecology, 47(2), 161–177. 10.1016/s0168-6496(03)00257-5 19712332

[ece311234-bib-0043] Kopprio, G. A. , Neogi, S. B. , Rashid, H. , Alonso, C. , Yamasaki, S. , Koch, B. P. , Gärdes, A. , & Lara, R. J. (2020). *Vibrio* and bacterial communities across a pollution gradient in the Bay of Bengal: Unraveling their biogeochemical drivers. Frontiers in Microbiology, 11, 594. 10.3389/fmicb.2020.00594 32351470 PMC7174592

[ece311234-bib-0044] Lai, J. , Jiang, F. , Ke, K. , Xu, M. , Lei, F. , & Chen, B. (2014). Nutrients distribution and trophic status assessment in the northern Beibu Gulf, China. Chinese Journal of Oceanology and Limnology, 32(5), 1128–1144. 10.1007/s00343-014-3199-y

[ece311234-bib-0045] Lawrence, R. P. , & William, J. W. (2001). Temperature and substrates as interactive limiting factors for marine heterotrophic bacteria. Aquatic Microbial Ecology, 23(2), 187–204. 10.3354/ame023187

[ece311234-bib-0046] Lehman, C. L. , & Tilman, D. (2000). Biodiversity, stability, and productivity in competitive communities. The American Naturalist, 156(5), 534–552. 10.1086/303402 29587515

[ece311234-bib-0047] Leibold, M. A. (1999). Biodiversity and nutrient enrichment in pond plankton communities. Evolutionary Ecology Research, 1(1), 73–95.

[ece311234-bib-0048] Li, L. , Pujari, L. , Wu, C. , Huang, D. , Wei, Y. , Guo, C. , & Sun, J. (2021). Assembly processes and Co‐occurrence patterns of abundant and rare bacterial community in the Eastern Indian Ocean. Frontiers in Microbiology, 12, 616956. 10.3389/fmicb.2021.616956 34456881 PMC8385211

[ece311234-bib-0049] Li, N. , Dong, K. , Jiang, G. , Tang, J. , Xu, Q. , Li, X. , & Adams, J. M. (2020). Stochastic processes dominate marine free‐living *Vibrio* community assembly in a subtropical gulf. FEMS Microbiology Ecology, 96(11), fiaa198. 10.1093/femsec/fiaa198 32990746

[ece311234-bib-0050] Liang, J. , Liu, J. , Wang, X. , Lin, H. , Liu, J. , Zhou, S. , & Zhang, X.‐H. (2019). Spatiotemporal dynamics of free‐living and particle‐associated *Vibrio* communities in the northern Chinese marginal seas. Applied and Environmental Microbiology, 85(9), e00217‐19. 10.1128/AEM.00217-19 30824453 PMC6495765

[ece311234-bib-0051] Liu, Y. , Du, J. , Xu, X. , Kardol, P. , & Hu, D. (2020). Microtopography‐induced ecohydrological effects alter plant community structure. Geoderma, 362, 114119. 10.1016/j.geoderma.2019.114119

[ece311234-bib-0052] Mansergh, S. , & Zehr, J. (2014). *Vibrio* diversity and dynamics in the Monterey Bay upwelling region. Frontiers in Microbiology, 5, 48. 10.3389/fmicb.2014.00048 24575086 PMC3921578

[ece311234-bib-0053] Matteucci, G. , Schippa, S. , Di Lallo, G. , Migliore, L. , & Thaller, M. C. (2015). Species diversity, spatial distribution, and virulence associated genes of culturable vibrios in a brackish coastal Mediterranean environment. Annals of Microbiology, 65(4), 2311–2321. 10.1007/s13213-015-1073-6

[ece311234-bib-0054] McCann, K. S. (2000). The diversity–stability debate. Nature, 405(6783), 228–233. 10.1038/35012234 10821283

[ece311234-bib-0055] McNaughton, S. J. (1988). Diversity and stability. Nature, 333(6170), 204–205. 10.1038/333204a0

[ece311234-bib-0056] Moriarty, D. (1998). Control of luminous *Vibrio* species in penaeid aquaculture ponds. Aquaculture, 164(1–4), 351–358. 10.1016/S0044-8486(98)00199-9

[ece311234-bib-0057] Muscarella, M. E. , Boot, C. M. , Broeckling, C. D. , & Lennon, J. T. (2019). Resource heterogeneity structures aquatic bacterial communities. The ISME Journal, 13(9), 2183–2195. 10.1038/s41396-019-0427-7 31053829 PMC6775984

[ece311234-bib-0058] Pontarp, M. , Canbäck, B. , Tunlid, A. , & Lundberg, P. (2012). Phylogenetic analysis suggests that habitat filtering is structuring marine bacterial communities across the globe. Microbial Ecology, 64(1), 8–17. 10.1007/s00248-011-0005-7 22286378 PMC3375428

[ece311234-bib-0059] Pruzzo, C. , Huq, A. , Colwell, R. R. , & Donelli, G. (2005). Pathogenic *Vibrio* species in the marine and estuarine environment. In S. Belkin & R. R. Colwell (Eds.), Oceans and health: Pathogens in the marine environment (pp. 217–252). Springer.

[ece311234-bib-0060] Riley, J. P. , & Chester, R. (2013). Chemical oceanography. Elsevier.

[ece311234-bib-0061] Shade, A. , Gregory Caporaso, J. , Handelsman, J. , Knight, R. , & Fierer, N. (2013). A meta‐analysis of changes in bacterial and archaeal communities with time. The ISME Journal, 7(8), 1493–1506. 10.1038/ismej.2013.54 23575374 PMC3721121

[ece311234-bib-0062] Shade, A. , Read, J. S. , Youngblut, N. D. , Fierer, N. , Knight, R. , Kratz, T. K. , & Stombaugh, J. (2012). Lake microbial communities are resilient after a whole‐ecosystem disturbance. The ISME Journal, 6(12), 2153–2167. 10.1038/ismej.2012.56 22739495 PMC3504957

[ece311234-bib-0063] Shi, Y. , Li, Y. , Xiang, X. , Sun, R. , Yang, T. , He, D. , & Chu, H. (2018). Spatial scale affects the relative role of stochasticity versus determinism in soil bacterial communities in wheat fields across the North China plain. Microbiome, 6(1), 27. 10.1186/s40168-018-0409-4 29402331 PMC5799910

[ece311234-bib-0064] Siboni, N. , Balaraju, V. , Carney, R. , Labbate, M. , & Seymour, J. R. (2016). Spatiotemporal dynamics of *Vibrio* spp. within the Sydney Harbour estuary. Frontiers in Microbiology, 7, 460. 10.3389/fmicb.2016.00460 27148171 PMC4829023

[ece311234-bib-0065] Sichert, A. , & Cordero, O. X. (2021). Polysaccharide‐bacteria interactions from the lens of evolutionary ecology. Frontiers in Microbiology, 12, 705082. 10.3389/fmicb.2021.705082 34690949 PMC8531407

[ece311234-bib-0066] Skoog, A. , & Benner, R. (1997). Aldoses in various size fractions of marine organic matter: Implications for carbon cycling. Limnology and Oceanography, 42(8), 1803–1813. 10.4319/lo.1997.42.8.1803

[ece311234-bib-0067] Smith, V. H. (2007). Microbial diversity‐productivity relationships in aquatic ecosystems. FEMS Microbiology Ecology, 62(2), 181–186. 10.1111/j.1574-6941.2007.00381.x 17868363

[ece311234-bib-0068] Stegen, J. C. , Lin, X. , Fredrickson, J. K. , Chen, X. , Kennedy, D. W. , Murray, C. J. , & Konopka, A. (2013). Quantifying community assembly processes and identifying features that impose them. The ISME Journal, 7(11), 2069–2079. 10.1038/ismej.2013.93 23739053 PMC3806266

[ece311234-bib-0069] Stevens, M. H. H. , & Carson, W. P. (2002). Resource quantity, not resource heterogeneity, maintains plant diversity. Ecology Letters, 5(3), 420–426.

[ece311234-bib-0070] Takemura, A. F. , Chien, D. M. , & Polz, M. F. (2014). Associations and dynamics of Vibrionaceae in the environment, from the genus to the population level. Frontiers in Microbiology, 5, 38. 10.3389/fmicb.2014.00038 24575082 PMC3920100

[ece311234-bib-0071] Thompson, F. L. , Iida, T. , & Swings, J. (2004). Biodiversity of Vibrios. Microbiology and Molecular Biology Reviews, 68(3), 403–431. 10.1128/MMBR.68.3.403-431.2004 15353563 PMC515257

[ece311234-bib-0072] Thompson, J. R. , Randa, M. A. , Marcelino, L. A. , Tomita‐Mitchell, A. , Lim, E. , & Polz, M. F. (2004). Diversity and dynamics of a North Atlantic coastal *Vibrio* community. Applied and Environmental Microbiology, 70(7), 4103–4110. 15240289 10.1128/AEM.70.7.4103-4110.2004 PMC444776

[ece311234-bib-0073] Tilman, D. , Reich, P. B. , & Knops, J. M. H. (2006). Biodiversity and ecosystem stability in a decade‐long grassland experiment. Nature, 441(7093), 629–632. 10.1038/nature04742 16738658

[ece311234-bib-0074] Torsvik, V. , Øvreås, L. , & Thingstad, T. F. (2002). Prokaryotic diversity–Magnitude, dynamics, and controlling factors. Science, 296(5570), 1064–1066. 10.1126/science.1071698 12004116

[ece311234-bib-0075] Travers, M. A. , Boettcher Miller, K. , Roque, A. , & Friedman, C. S. (2015). Bacterial diseases in marine bivalves. Journal of Invertebrate Pathology, 131, 11–31. 10.1016/j.jip.2015.07.010 26210496

[ece311234-bib-0076] Urdaci, M. C. , Stal, L. J. , & Marchand, M. (1988). Occurrence of nitrogen fixation among *Vibrio* spp. Archives of Microbiology, 150(3), 224–229. 10.1007/BF00407784

[ece311234-bib-0077] Vellend, M. (2003). Island biogeography of genes and species. The American Naturalist, 162(3), 358–365. 10.1086/377189 12970843

[ece311234-bib-0078] Vellend, M. (2010). Conceptual synthesis in community ecology. The Quarterly Review of Biology, 85(2), 183–206. 10.1086/652373 20565040

[ece311234-bib-0079] Vezzulli, L. , Pezzati, E. , Moreno, M. , Fabiano, M. , Pane, L. , Pruzzo, C. , & The VibrioSea, C. (2009). Benthic ecology of *Vibrio* spp. and pathogenic *Vibrio* species in a coastal Mediterranean environment (La Spezia Gulf, Italy). Microbial Ecology, 58(4), 808–818. 10.1007/s00248-009-9542-8 19543938

[ece311234-bib-0080] Wagg, C. , Roscher, C. , Weigelt, A. , Vogel, A. , Ebeling, A. , de Luca, E. , & Schmid, B. (2022). Biodiversity–stability relationships strengthen over time in a long‐term grassland experiment. Nature Communications, 13(1), 7752. 10.1038/s41467-022-35189-2 PMC9751076 36517483

[ece311234-bib-0081] Wang, X. , Liu, J. , Li, B. , Liang, J. , Sun, H. , Zhou, S. , & Zhang, X.‐H. (2019). Spatial heterogeneity of *Vibrio* spp in sediments of Chinese marginal seas. Applied and Environmental Microbiology, 85(10), e03064‐18. 10.1128/AEM.03064-18 30877118 PMC6498182

[ece311234-bib-0082] Wang, X. , Liu, J. , Zhao, W. , Liu, J. , Liang, J. , Thompson, F. , & Zhang, X.‐H. (2022). Fine‐scale structuring of planktonic *Vibrio* spp in the Chinese marginal seas. Applied and Environmental Microbiology, 88(23), e01262‐22. 10.1128/aem.01262-22 36346224 PMC9746320

[ece311234-bib-0083] Wang, Y. , Du, J. , Pang, Z. , Liu, Y. , Xue, K. , Hautier, Y. , & Ji, B. (2022). Unimodal productivity–biodiversity relationship along the gradient of multidimensional resources across Chinese grasslands. National Science Review, 9(12), nwac165. 10.1093/nsr/nwac165 36519072 PMC9743175

[ece311234-bib-0084] Westrich, J. R. (2015). Consilience of iron in the ecology of Vibrio bacteria. University of Georgia.

[ece311234-bib-0085] Worm, B. , Lotze, H. K. , Hillebrand, H. , & Sommer, U. (2002). Consumer versus resource control of species diversity and ecosystem functioning. Nature, 417(6891), 848–851. 10.1038/nature00830 12075351

[ece311234-bib-0086] Wright, A. C. , Hill, R. T. , Johnson, J. A. , Roghman, M. C. , Colwell, R. R. , & Morris, J. G. (1996). Distribution of *Vibrio vulnificus* in the Chesapeake Bay. Applied and Environmental Microbiology, 62(2), 717–724. 10.1128/aem.62.2.717-724.1996 8593075 PMC167840

[ece311234-bib-0087] Xu, W. , Gong, L. , Yang, S. , Gao, Y. , Ma, X. , Xu, L. , & Luo, Z. (2020). Spatiotemporal dynamics of *Vibrio* communities and abundance in Dongshan Bay, South of China. Frontiers in Microbiology, 11, 575287. 10.3389/fmicb.2020.575287 33324364 PMC7726330

[ece311234-bib-0088] Xu, W. , Lin, W. , Wang, Z. , Gao, Y. , Luo, Y. , Grossart, H.‐P. , & Luo, Z. (2021). Disentangling the abundance and structure of vibrio communities in a semi‐enclosed bay with mariculture (Dongshan Bay, southern China). Computational and Structural Biotechnology Journal, 19, 4381–4393. 10.1016/j.csbj.2021.07.040 34429854 PMC8365367

[ece311234-bib-0089] Xun, W. , Liu, Y. , Li, W. , Ren, Y. , Xiong, W. , Xu, Z. , & Zhang, R. (2021). Specialized metabolic functions of keystone taxa sustain soil microbiome stability. Microbiome, 9(1), 35. 10.1186/s40168-020-00985-9 33517892 PMC7849160

[ece311234-bib-0090] Yang, X. , Cheng, J. , Franks, A. E. , Huang, X. , Yang, Q. , Cheng, Z. , & He, Y. (2023). Loss of microbial diversity weakens specific soil functions, but increases soil ecosystem stability. Soil Biology and Biochemistry, 177, 108916. 10.1016/j.soilbio.2022.108916

[ece311234-bib-0091] Yodzis, P. (1981). The stability of real ecosystems. Nature, 289(5799), 674–676. 10.1038/289674a0

[ece311234-bib-0092] Zakem, E. J. , Al‐Haj, A. , Church, M. J. , van Dijken, G. L. , Dutkiewicz, S. , Foster, S. Q. , & Follows, M. J. (2018). Ecological control of nitrite in the upper ocean. Nature Communications, 9(1), 1–13. 10.1038/s41467-018-03553-w PMC5865239 29572474

[ece311234-bib-0093] Zeng, J. , Lin, Y. , Zhao, D. , Huang, R. , Xu, H. , & Jiao, C. (2019). Seasonality overwhelms aquacultural activity in determining the composition and assembly of the bacterial community in Lake Taihu, China. Science of the Total Environment, 683, 427–435. 10.1016/j.scitotenv.2019.05.256 31141745

[ece311234-bib-0094] Zhang, X. , Lin, H. , Wang, X. , & Austin, B. (2018). Significance of vibrio species in the marine organic carbon cycle—A review. Science China Earth Sciences, 61, 1357–1368. 10.1007/s11430-017-9229-x

[ece311234-bib-0095] Zhou, J. , & Ning, D. (2017). Stochastic community assembly: Does it matter in microbial ecology? Microbiology and Molecular Biology Reviews, 81(4), e00002‐17. 10.1128/MMBR.00002-17 29021219 PMC5706748

[ece311234-bib-0096] Zhou, K. , Tian, K. Y. , Liu, X. Q. , Liu, W. , Zhang, X. Y. , Liu, J. Y. , & Sun, F. (2021). Characteristic and Otopathogenic analysis of a Vibrio alginolyticus strain responsible for chronic otitis externa in China. Frontiers in Microbiology, 12, 750642. 10.3389/fmicb.2021.750642 34975783 PMC8718755

