# Supplementary material for: Resource diversity disturbs marine Vibrio diversity and community stability, but loss of Vibrio diversity enhances community stability
Source: Ecol Evol. 2024 Apr 19;14(4):e11234. doi: 10.1002/ece3.11234 (PMC11027015; doi:10.1002/ece3.11234)
Supplement: Supplementary file 1 — Figures S1–S4. [file ECE3-14-e11234-s001.docx]

**Supplementary Information-2 (Fig. S1-S4)**

**Resource Diversity Disturbs Marine *Vibrio* Diversity and Community Stability, but Loss of *Vibrio* Diversity Enhanc-es Community Stability**

Xinyi Qin^1,2^, Huaxian Zhao^2^, Pengbin Wang^3^, Shu Yang^2^, Nengjian Liao^4^, Jiongqing Huang^5^, Xiaoli Li^5^, Qing He^1^, Rajapakshalage Thashikala Nethmini^1^, Gonglingxia Jiang^1^, Shiying He^2^, Qingxiang Chen^1^, Ke Dong^6^, Qinghua Hou^1**^, Nan Li^1*^

^1^ Laboratory for Coastal Ocean Variation and Disaster Prediction, College of Ocean and Meteorology; Key Laboratory of Climate, Resources and Environment in Continental Shelf Sea and Deep Sea of Department of Education of Guangdong Province, Guangdong Ocean University, Zhanjiang 524088, China

^2^ Key Laboratory of Environment Change and Resources Use in Beibu Gulf, Ministry of Education, Nanning Normal University, Nanning, 530001, China

^3^ Key Laboratory of Marine Ecosystem Dynamics, Second Institute of Oceanography, Ministry of Natural Re-sources, Hangzhou, 310012, China

^4^ College of Environmental Science and Engineering, Guilin University of Technology, Guilin, 541006, China

^5^ School of Agriculture, Ludong University, Yantai 264025, China

^6^ Department of Biological Sciences, Kyonggi University, 154-42, Gwanggyosan-ro, Yeongtong-gu, Suwon-si, Gyeonggi-do 16227, South Korea

*** Correspondence:***Corresponding Author: Nan Li

nli0417@163.com

**Corresponding Author: Qinghua Hou

huaqingh@163.com


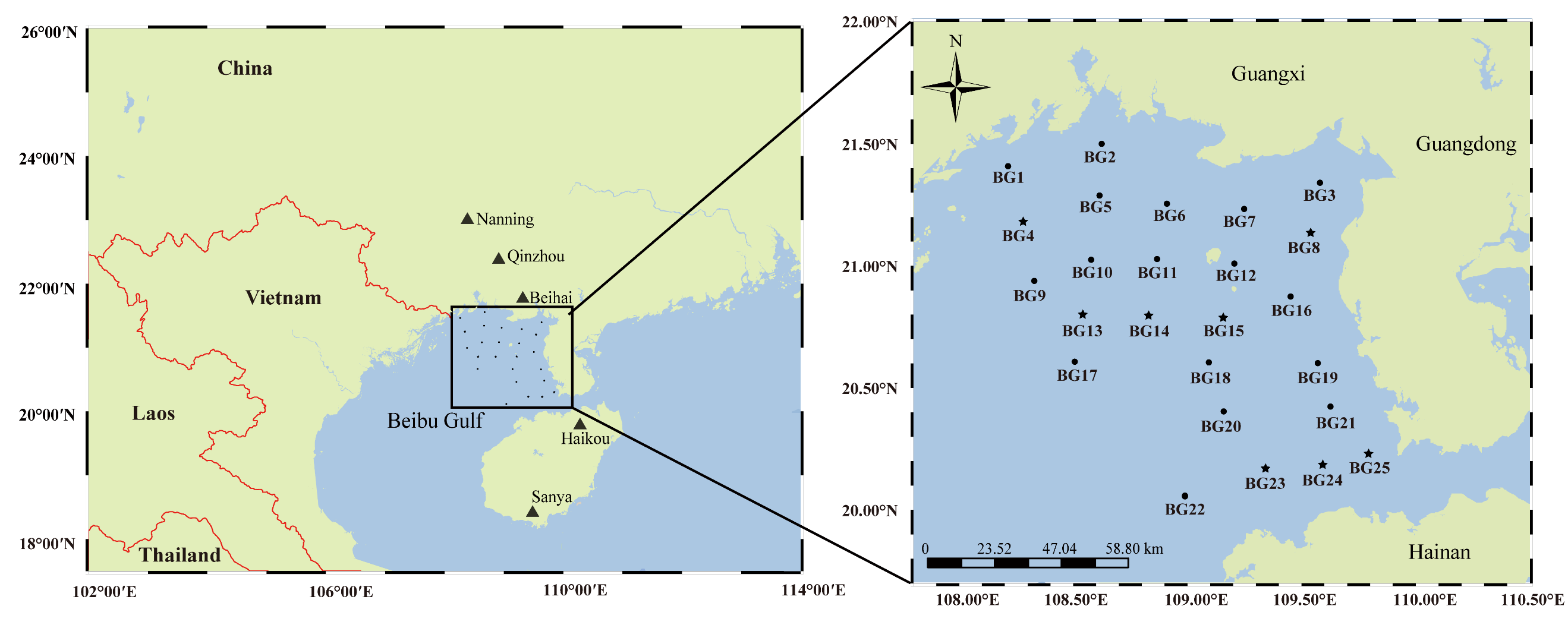


**Figure S1** Geographic map of sampling sites. Black dots mark: seawater and sediment sampling sites. Black star mark: sediment sampling sites.


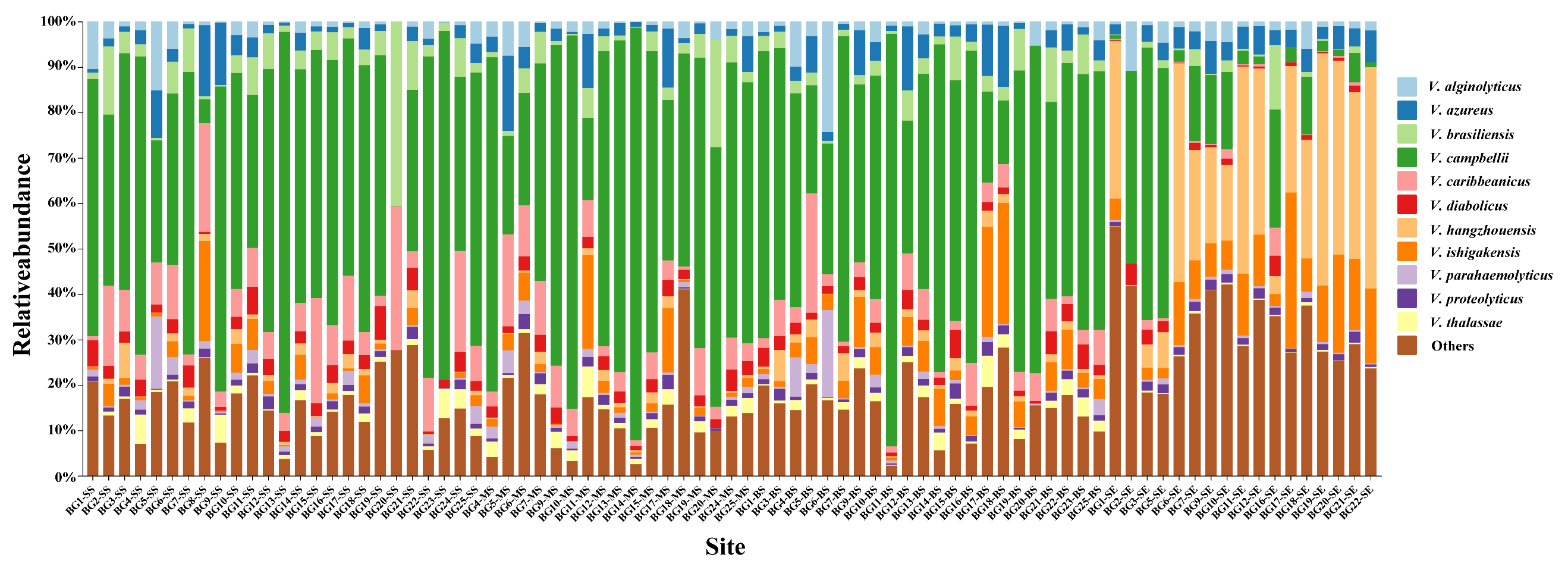


**Figure S2** Relative abundances of 11 most abundant marine *Vibrio* species in different depth and habitats of each sampling sites.


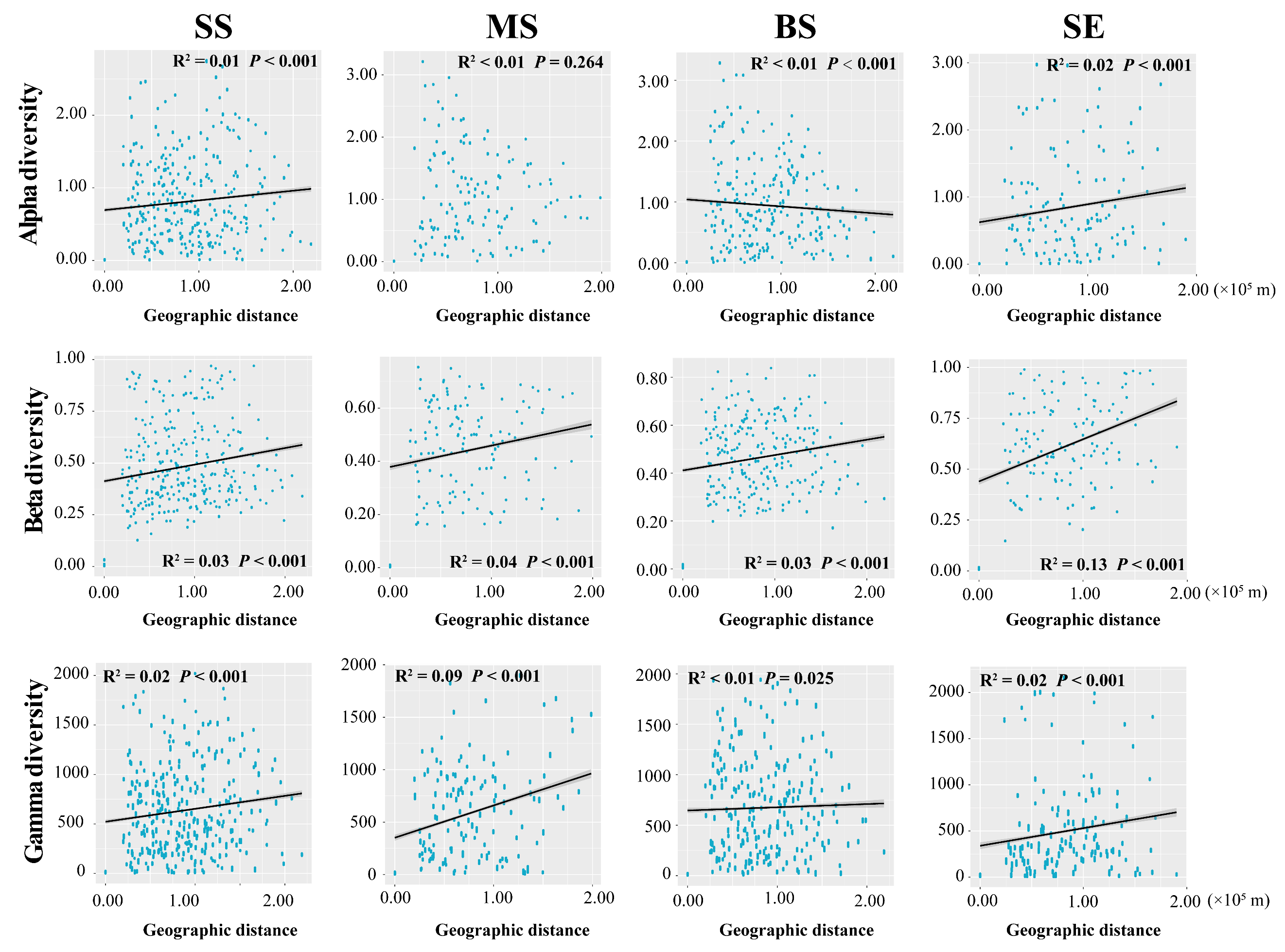


**Figure S3** Linear regressions for geographic distance associated with marine *Vibrio* alpha diversity, beta diversity, and gamma diversity.


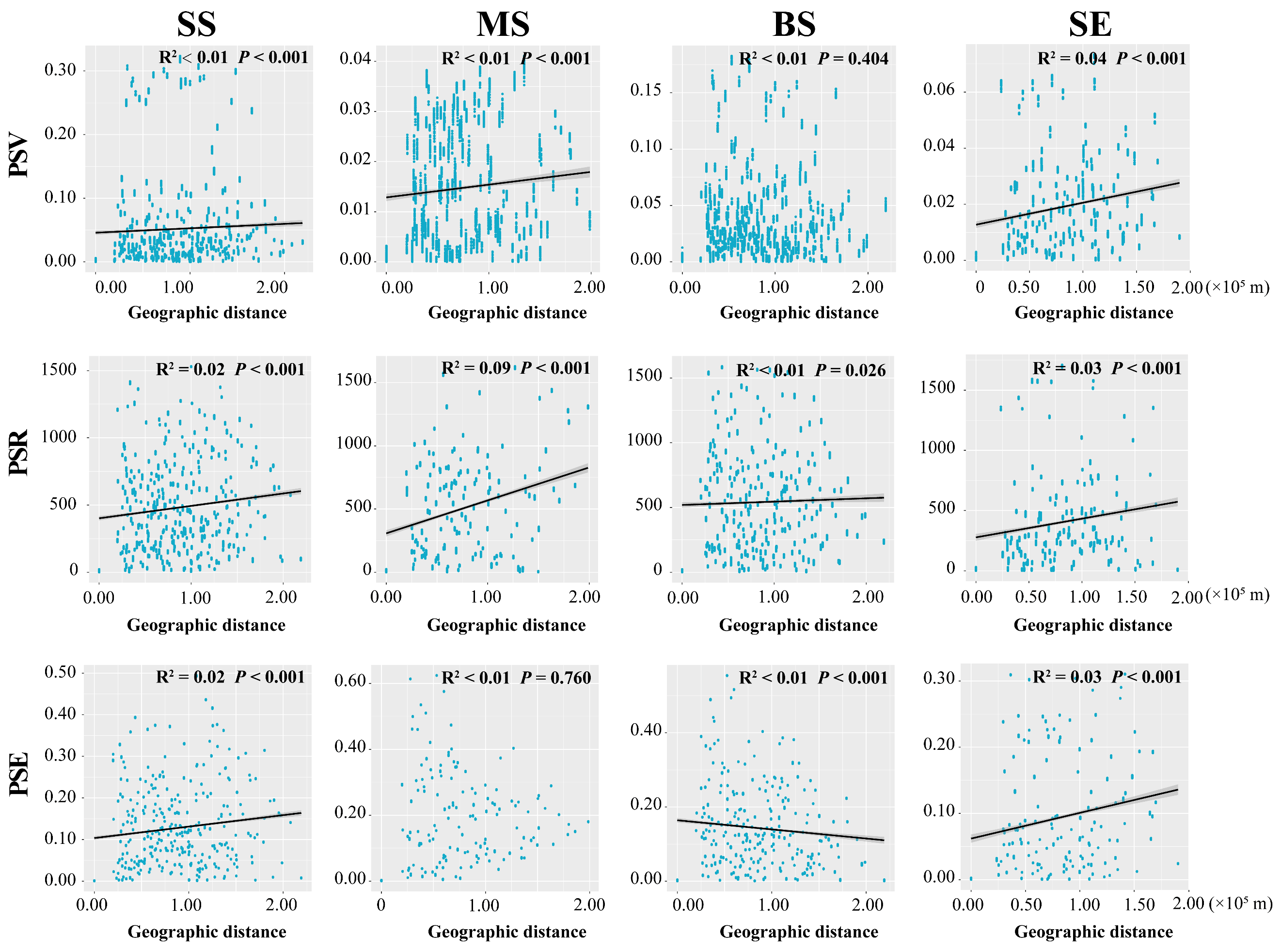


**Figure S4** Linear regressions for geographic distance associated with PSE, PSR, and PSV.
